# Blocking the SIRPα-CD47 axis promotes macrophage phagocytosis of exosomes derived from visceral adipose tissue and improves inflammation and metabolism in mice

**DOI:** 10.1186/s12929-025-01124-y

**Published:** 2025-02-28

**Authors:** Yun-kai Lin, Yu-fei Pan, Tian-yi Jiang, Yi-bin Chen, Tai-yu Shang, Meng-you Xu, Hui-bo Feng, Yun-han Ma, Ye-xiong Tan, Hong-yang Wang, Li-wei Dong

**Affiliations:** 1 International Cooperation Laboratory On Signal Transduction, Eastern Hepatobiliary Surgery Hospital, Naval Medical University, Shanghai, China; 2 Oncology Pharmacy Laboratory, National Center for Liver Cancer, Shanghai, China; 3 State Key Laboratory of Oncogenes and Related Genes, Shanghai Cancer Institute, Renji Hospital, Shanghai Jiaotong University School of Medicine, Shanghai, China

**Keywords:** Adipose tissue, Exosomes, miRNAs, Obesity, Macrophages, SIRPα-CD47

## Abstract

**Background:**

Adipose tissue plays a pivotal role in systemic metabolism and maintaining bodily homeostasis. Exosomes from adipose tissues, known as AT-Exos, are recognized as important messengers in the communication between adipose tissue and other organs. Despite this, the alterations in exosome composition and the functional disparities among depot-specific AT-Exos in obesity remain elusive.

**Methods:**

In this work, we utilized lipidomics and microRNA (miRNA) sequencing to elucidate the lipid and miRNA profiles of AT-Exos in a diet-induced obesity model. We identified obesity-related miRNAs in AT-Exos and further explored their mechanisms using gain- and loss-of-function experiments. To evaluate the metabolic effects of AT-Exos on adipocytes, we conducted RNA-sequencing (RNA-seq) and confirmed our findings through Quantitative Real-time PCR (qPCR) and Western bolt analyses. Meanwhile, a mouse model with intraperitoneal injections was utilized to validate the role of exosomes derived from visceral white adipose tissue (vWAT-Exos) in obesity progression in vivo. Finally, we explored potential therapeutic intervention strategies targeting AT-Exos, particularly focusing on modulating the SIRPα-CD47 axis to enhance macrophage phagocytosis using Leptin-deficient (ob/ob) mice and SIRPα knock-out mice.

**Results:**

Our study revealed that obesity-related metabolism affects the biological processes of AT-Exos, with depot-specific secretion patterns. In obesity, the lipidome profile of AT-Exos was significantly altered, and diet can modify the miRNA content and function within these exosomes, influencing lipid metabolism and inflammatory pathways that contribute to metabolic dysregulation. Specifically, we identified that miR-200a-3p and miR-200b-3p promoted lipid accumulation in 3T3L1 cells partly through the PI3K/AKT/mTOR pathway. RNA-Seq analysis revealed that AT-Exos from different fat depots exerted distinct effects on adipocyte metabolism, with obese vWAT-Exos being notably potent in triggering inflammation and lipid accumulation in diet-induced obesity. Additionally, we found that inhibiting the SIRPα-CD47 axis can mitigate metabolic disorders induced by obese vWAT-Exos or ob/ob mice, partly due to the enhanced clearance of vWAT-Exos. Consistent with this, SIRPα-deficient mice exhibited a reduction in vWAT-Exos and displayed greater resistance to obesity.

**Conclusions:**

This study elucidates that diet-induced obesity altered the lipid and miRNA profiles of AT-Exos, which involved in modulating adipocyte inflammation and metabolic balance. The SIRPα-CD47 axis emerges as a potential therapeutic target for obesity and its associated complications.

**Supplementary Information:**

The online version contains supplementary material available at 10.1186/s12929-025-01124-y.

## Background

Adipose tissue (AT) can be classified into two main types: white adipose tissue (WAT), which is the primary site of energy storage, and brown adipose tissue (BAT), known for its energy expenditure capabilities mediated by the activation of uncoupling protein 1 (UCP1) [1]. Increasing evidence highlights the functional heterogeneity of WAT, which is characterized by its diverse metabolic functions and its ability to communication with other tissues through the secretion of bioactive molecules such as peptides, lipids, and nucleic acids, all of which influence systemic metabolic processes [2, 3]. Anatomically, WAT is divided into two principal depots: visceral white adipose tissue (vWAT) and subcutaneous adipose tissue (SAT). Accumulation of vWAT is associated with insulin resistance (IR) and an increased risk of metabolic disorders, while the buildup of SAT, such as inguinal white adipose tissue (iWAT), is considered benign and may even confer protection against metabolic syndrome [4, 5]. Studies have shown that individuals with subcutaneous obesity exhibit a reduced cardiovascular risk, irrespective of the presence of visceral obesity [6, 7]. The transplantation of SAT into mice enhances glucose metabolism, indicating that these benefits are due to intrinsic cellular differences rather than their anatomical position [8]. Both vWAT and SAT function as endocrine organs, and their distinct metabolic properties help maintain the body's energy homeostasis. However, the underlying mechanisms of the heterogeneity of AT and its distinct effects on metabolism remain to be fully unveiled.

Endocrine functions of AT have been extensively investigated since the 1980s, and a variety of adipokines, including leptin and adiponectin, have been identified that affect metabolism and energy homeostasis [9–11]. Recently, extracellular vesicle (EV), especially AT-secreted exosomes, have been recognized as integral components of the WAT secretome, facilitating communication between metabolic organs [12]. Exosomes are nanosized EVs (30–160 nm diameter) with lipid bilayer membranes and contain various contents, including lipids, miRNAs and proteins, all of which are widely involved in signaling pathways and genetic information processes [13]. Given the pivotal role of WAT in obesity, AT-Exos may harbor valuable insights into the detrimental effects of obesity and are capable of modulating the signaling pathways in target cells. Some studies have reported that AT-Exos can directly disrupt insulin signaling in liver and muscle cells [14] and enhance lipid synthesis by elevating lipogenic enzyme levels [15, 16]. Furthermore, BAT-Exos have been shown to mitigate metabolic syndrome in obese mice by modulating catalytic processes that boost oxygen consumption in recipient cells [17]. These findings collectively highlight the emerging potential of AT-Exos as novel adipokines that contribute to either the maintenance or disruption of metabolic homeostasis. The intricate composition of AT-Exos and their diverse metabolic regulatory capabilities are fueling increasing interest in their potential as targets for metabolic interventions. Adipose tissue is a connective tissue composed mostly of adipocytes and a small fraction of stromal and immune cells [18]. Adipose tissue macrophages (ATMs) account for the most enriched immune cells in obese AT (from 10% in lean states up to 40% in obese states) [19]. ATMs have proven to be the primary executors responsible for the development of IR since the main source of proinflammatory cytokines in AT is ATMs [20]. AT-Exos from obese mice have been shown to activate ATMs, leading to an increase in the production of proinflammatory cytokines [21]. Additionally, adipose tissue-derived EVs, especially exosomes, have been found to induce primary monocytes to acquire phenotypes characteristic of ATM [22]. Notably, adipocyte-derived exosomes are also shown to contain lipid droplets, and these exosomes are actively phagocytosed by ATMs [23]. Therefore, exosomes released by adipose tissue can act as a means of communication between adipose tissues and macrophages.

Signaling regulatory protein α (SIRPα), as a ligand for CD47, is an immune checkpoint expressed on macrophages, dendritic cells (DCs) and neutrophils. SIRPα-CD47 axis provides inhibitory signaling in monocytes and macrophages, attenuating phagocytosis and inflammation [24, 25]. However, the involvement of this pathway in fatty acid-induced inflammatory responses has not been elucidated. Given that AT-Exos are the critical biological mediators between adipocytes and ATMs in adipose tissue [26], it is of great interest to explore whether blocking SIRPα-CD47 signaling could affect the homeostasis of AT-Exos.

In this study, we revealed that diet-induced obesity differently altered the lipid and miRNA profiles of exosomes from iWAT, vWAT and BAT. Based on RNA-seq analysis, we found AT-Exos exerted specific effects on inflammation and glycolipid metabolism in adipocytes in a depot-dependent manner. Notably, obese iWAT-Exos and BAT-Exos exhibited similar functions in increasing lipid utilization. In contrast, obese vWAT-Exos were identified to markedly promote lipid synthesis and inflammation, exacerbating glucose intolerance and hepatic steatosis. These findings indicated the critical roles of vWAT-Exos in the pathogenesis of obesity and its associated metabolic disorders. Particularly, miRNA-200a-3p and miRNA-200b-3p, which were upregulated in vWAT-Exos, promoted lipid accumulation in adipocytes via the PI3K-AKT-mTOR pathway. Obese vWAT-Exos were also observed to activate ATMs, manifested as a significant increase in proinflammatory cytokines and chemokines. In addition, we found that blocking SIRPα-CD47 enhances macrophage phagocytosis of vWAT-Exos and thus alleviates metabolic disorders and tissue inflammation in obese mice. These insights reveal a novel exosome-mediated mechanism of crosstalk between adipocytes and macrophages, offering new avenues for therapeutic intervention in obesity-associated metabolic syndrome.

## Methods

### Mice

SIRPα^loxp/loxp^ mice were generated by the Model Animal Research Center of Nanjing University. C57BL/6 mice (JAX: 000664) and Lyz-Cre mice (JAX: 004781) were obtained from Jackson Laboratory at ages ranging from 4 to 12 weeks old. Leptin-deficient (ob/ob) mice aged 4–5 weeks were purchased from GemPharmatech Co., Ltd. (Nanjing, China). Mice with macrophage-specific deletion of SIRPα (SIRPα^ΔMac^) were generated in the F2 generation of animals from the intercrossing of Lyz-Cre and SIRPα^loxp/loxp^ mice. To genotype animals, DNA was isolated from tail lysates, and PCR was performed using the PCR primers listed in Table S1. All mice were randomly assigned to experimental groups (3–5 male mice per cage). Mice were sacrificed using CO_2_, and all fat pads were immediately excised for further processing and analysis. All animal work was approved by the Institutional Animal Care and Use Committees of Naval Medical University.

### Primary cells and cell line cultures

To obtain SVF cells, epididymal fat tissues were collected, cleaned, weighed, and cut into small pieces using scissors. The tissue pieces were then incubated in PBS containing 0.2% collagenase type I (Sigma-Aldrich, C1-BIOC) for 1 h at 37 °C with gentle shaking. After incubation and passed through a 70 μm filter (BD Falcon, 352350), the SVF cells were collected by centrifugation at 500 × g for 5 min at 4 °C. The cell pellet was washed with PBS before counting the number of cells. To obtain spleen cells, the tissue was cut into small particles with scissors, and red blood cells (RBCs) were lysed with ammonium chloride lysing buffer. Cells were passed through a 0.22 μm nylon membrane (Dakewe Biotech) before use. To obtain mouse primary Bone marrow-derived macrophages (BMDMs), bone marrow cells were harvested from the femurs and tibias of 5-week-old wild-type B6 mice. After lysis of RBCs, cells were propagated in bacterial petri dishes for 7 days in RPMI-1640 medium supplemented with 10% FBS (Gibco, A5670701), GlutaMAX (Gibco, 35050061), penicillin–streptomycin and recombinant mouse M-CSF (15 ng/ml, Peprotech, 315–02). On day 7, cells were harvested.

3T3-L1 cells or SVF cells were maintained in Dulbecco’s Modified Eagle Medium (DMEM, BasalMedia Technologies, L110KJ) supplemented with penicillin–streptomycin and 10% newborn calf serum (NCS, Gibco, 26010074). For differentiation into adipocytes, the medium was replaced with DMEM containing 1% penicillin–streptomycin, 10% FBS, 10 μg/ml insulin (Sigma-Aldrich, I2643), 0.5 mM IBMX (Selleck, S5836) and 1 μM dexamethasone (Selleck, S1322) starting 2 days after the cells reached confluence (day 0). On day 3, the medium was replaced every other day with complete DMEM containing insulin (10 μg/ml). Fully differentiated adipocytes (days 8–10) were used for assays [27, 28]. OP9 cells were purchased from Wuhan Pricella Biotechnology. Cells were maintained in Minimum Essential Medium Alpha (MEM-α, (Invitrogen, 12561056) with 20% FBS, 26 mM sodium bicarbonate, 100 U/ml penicillin, 100 μg/ml streptomycin, 0.25 μg/ml amphotericin B. Cells were plated in 24-well plates at 50,000 cells per well in 500 μl medium and incubated for 4 days. Induction and maintenance of adipogenesis and treatment were as described for 3T3L1 cells.

For the exosome stimulation assay, approximately 2 × 10^12^ exosomes from adipose tissue, liver or muscle were added to fully differentiated 3T3-L1 adipocytes, SVF adipocytes, OP9 cells or BMDMs in 6-well cell culture plates. After incubation for 48 h at 37 °C, cells were washed and lysed with TRIzol Reagent (Invitrogen, 15596026CN) for RNA extraction and further analysis. Each type of AT-Exos was assessed at least in triplicate.

### Isolation and analysis of AT-Exos

Adipose tissue was taken from LFD-fed or HFD-fed mice for exosome isolation according to previous studies with minor modifications [23]. Tissues were fully shredded and cultured in MEM-α (Invitrogen, 12561056) for 1 h. After washing and centrifugation with MEM-α, the tissue mass was cultured in MEM-α for 48 h. The sample was passed through a 70 μm filter (BD Falcon, 352350), and conditioned medium was collected. The harvested medium was centrifuged at 300 × g for 10 min, 2000 × g for 20 min and 10,000 × g for 30 min to remove cells and debris. The supernatant was ultracentrifuged at 120,000 × g for 2 h using an Optima MAX-XP (Beckman Coulter). The exosome pellet was resuspended in PBS and ultracentrifuged at 120,000 × g for 2 h again. Finally, the exosome pellet was resuspended in PBS for transmission electron microscopy (TEM, FEI Company) and quantified by ZetaView PMX110 (Particle Metrix), in serum-free medium for cell-based assays, lysed with 100 μl of RIPA buffer for protein extraction or lysed with TRIzol Reagent (Invitrogen, 15596026CN) for RNA extraction. Exosome release from lean or obese tissue expressed per gram of tissue, as measured by Nanoparticle Tracking Analysis.

Liver or muscle tissue was collected and cut into small pieces using scissors. The tissue pieces were then incubated in MEM-α containing 1 mg/ml collagenase type II (Sigma-Aldrich, C2-BIOC) for 1 h at 37 °C with gentle shaking. After washing and centrifugation with MEM-α, the tissue mass was cultured in MEM-α for 48 h. The sample was passed through a 70 μm filter, and conditioned medium was collected. The isolation and analysis of exosomes were as described for adipose tissues.

### In vivo studies

8–10 weeks old male littermate SIRPα^loxp/loxp^ and SIRPα^ΔMac^ mice were given a high-fat diet (HFD, TROPHIC Animal Feed High-Tech Co., Ltd., TP23400) for 16 weeks to induce obesity and mice on a low-fat diet (LFD, TP23402) served as lean controls. Body weight, food and water consumption were monitored daily in all groups. For the SIRPα-CD47 axis blocking study, 8- to 10-week-old male B6 mice were intraperitoneally injected every other day with lgG2a (10 mg/kg, Bio X cell, BE0089) or neutralizing antibodies (NAs) targeting CD47 (NA-CD47) (10 mg/kg, Bio X cell, BE0270) for 4 weeks. For the treatment of obese vWAT-Exo-induced metabolic disorders, 8- to 10-week-old male B6 mice were administered obese vWAT-Exos via tail vein injection (30 μg/mouse, once every 3 days) [21, 29], together with administration of lgG or NA-CD47 (10 mg/kg, once every 3 days, Bio X cell) by intraperitoneal injection for 8 weeks. For the treatment of ob/ob mice, 5-week-old ob/ob mice were divided into two groups and treated with lgG or NA-CD47 (20 mg/kg, 3 time a week, Bio X cell) by intraperitoneal injection for 8 weeks.

### Metabolic parameters

Serum alanine aminotransferase (ALT), triacylglycerol (TG), total cholesterol (TC), high-density lipoprotein cholesterol (HDL-c) and low-density lipoprotein cholesterol (LDL-c) were detected with an automatic biochemical analyzer (Mindray Bio-Medical Electronics Co., Ltd.). Serum leptin (EK297), insulin (PI602), and cytokine levels, including interleukin-6 (IL-6, EK206), interleukin-1β (IL-1β, EK201B) and tumor necrosis factor-alpha (TNF-α, EK282HS) in the cell culture supernatant, were measured after the intervention using commercial kits (Multi-sciences Biotech Co., Ltd). For the intraperitoneal glucose tolerance test (IPGTT), the mice were fasted for 16 h. For the intraperitoneal insulin tolerance test (IPITT), the mice were fasted for 4 h. Glucose at 2 g/kg or insulin at 0.75 U/kg in saline were administered via intraperitoneal injection, and blood glucose was measured before and at 15 min, 30 min, 60 min, 90 min and 120 min post-injection using a glucometer (Bayer Corporation). For metabolic cage analysis, mice were individually housed in metabolic chambers (Techniplast E-chiller) with free access to food and water and were maintained on a 12-h:12-h dark: light cycle. Mice were acclimatized in metabolic chambers for 24 h before initiation of data collection. The volume of oxygen consumption (VO_2_) and carbon dioxide production (VCO_2_) were determined every 20 min for a period of 3 days. Food intake and locomotion activity were also determined.

### RNA isolation and PCR

White and brown fat tissues were snap-frozen in liquid nitrogen. Total RNA was isolated and purified using TRIzol Reagent (Invitrogen, 15596026CN) and reverse transcription was performed with the M-MLV reserve transcriptase instructions (Promega, M1705). Levels of cDNA were quantified by qPCR using SYBR Green Supermix (Roche, 04887352001) with gene-specific primers, and the results were normalized to β-actin or 36B4 control. Each sample was assessed at least in triplicate. The primers used for real-time PCR are listed in Table S2.

### RNA-seq and data analysis

We performed 2 × 150-bp paired-end sequencing (PE150) using Illumina NovaSeq 6000 (LC-Bio Technology Co., Ltd.) as previously described [30]. Briefly, total RNA of 3T3-L1 cells or SVF cells was isolated using TRIzol reagent (Invitrogen, 15596026CN). The RNA integrity was assessed by a Bioanalyzer 2100 (Agilent) with RIN > 7.0 and confirmed by electrophoresis with denaturing agarose gel. cDNA was then generated by SuperScript™ II Reverse Transcriptase (Invitrogen, 18064071), which was next used to synthesize U-labeled second-stranded DNA with E. coli DNA polymerase I (NEB, M0209), RNase H (NEB, M0297L) and dUTP Solution (Thermo Fisher Scientific, R0133). An A-base is then added to the blunt ends of each strand, preparing them for ligation to the indexed adapters. Each adapter contains a T-base overhang for ligating the adapter to the A-tailed fragmented DNA. Single- or dual-index adapters were ligated to the fragments, and size selection was performed with AMPureXP beads. After the heat-labile UDG enzyme (NEB, M0280L) treatment of the U-labeled second-stranded DNAs, the ligated products were amplified with PCR.

Fastp software (https://github.com/OpenGene/fastp) was used to remove reads that contained adapter contamination and low-quality bases with default parameters and to verify sequence quality. HISAT2 (https://ccb.jhu.edu/software/hisat2) was used to map the reads to the reference genome of Homo sapiens GRCh381. The mapped reads of each sample were assembled using StringTie (https://ccb.jhu.edu/software/stringtie). Then, all transcriptomes from all samples were merged to reconstruct a comprehensive transcriptome using gffcompare (https://github.com/gpertea/gffcompare/). After the final transcriptome was generated, StringTie was used to determine the expression level for mRNAs by calculating FPKM (fragments per kilobase of exon model per million mapped fragments). The differentially expressed mRNAs were selected with fold change > 2 or fold change < 0.5 and with the parametric F test comparing nested linear models (p value < 0.05) by the R package edgeR. Pathway analysis was employed to analyze the main functions of the differentially expressed genes according to the Gene Ontology (GO) database or Kyoto Encyclopedia of Genes and Genomes (KEGG) database. Gene set enrichment analysis (GSEA) was performed for gene lists sorted by the log (fold change).

### Lipidomics studies

Purified AT-Exos were thawed on ice, and metabolites were extracted with 50% methanol buffer. The details were as follows: 20 μl of AT-Exos solution was extracted with 120 μl of precooled 50% methanol, vortexed for 1 min, and incubated at room temperature for 10 min; the extraction mixture was then stored overnight at − 20 °C. After centrifugation at 4000 × g for 20 min, the supernatants were transferred into new 96-well plates. The samples were stored at − 80 °C prior to liquid chromatography-mass spectrometry (LC–MS) analysis. In addition, pooled quality control (QC) samples were also prepared by combining 10 μl of each extraction mixture. All samples were acquired by the LC–MS system following the manufacturer’s instructions.

XCMS software (SCIEX) was used to perform the acquired MS data pretreatments. LC–MS raw data files were converted into mzXML format and then processed by the XCMS, CAMERA and metaX toolbox implemented with R software. The online Kyoto Encyclopedia of Genes and Genomes (KEGG) and Human Metabolome Database were used to annotate the metabolites by matching the exact molecular mass data (m/z) of samples with those from the database. An in-house fragment spectrum library of metabolites was used to validate the metabolite identification. The intensity of peak data was further preprocessed by metaX. Student’s t tests were conducted to detect differences in metabolite concentrations between the 2 phenotypes. The *P* value was adjusted for multiple tests using the False discovery rate (FDR, Benjamini-Hochberg). Supervised Partial Least-Squares Discriminant Analysis (PLS-DA) was conducted through metaX to discriminate the different variables between groups. The VIP (variable importance in projection) value was calculated. A VIP cutoff value of 1.0 was used to select important features.

### miRNA sequencing and targeted genes prediction

Total RNA was isolated and purified following the manufacturer’s procedure. The RNA amount and purity of each sample was evaluated using NanoDrop ND-1000 (NanoDrop, Wilmington, DE). The RNA fragment integrity was evaluated through Bioanalyzer 2100 (Agilent, CA). The sequencing strategy was Single-end 50 bp for Illumina Hiseq 2500 following the vendor’s recommended protocol.

To predict the genes targeted by most aboundant miRNAs, two computational target prediction algorithms (TargetScan (5.0) and Miranda (3.3a), TargetScan_score ≥ 50 and miranda_Energy < − 10) were used to identify miRNA binding sites. The data predicted by both algorithms were combined and the overlaps were calculated. GO/KEGG enrichment analysis provides all GO terms/KEGG pathways that significantly enriched in miRNA target genes comparing to the genome background.

### Cell transfection

miR-200a-3p and miR-200b-3p mimics/inhibitors and their respective negative controls were purchased from Ribobio Co. (Guangzhou, China). Transfection was performed using JetPRIME reagent (Polyplus, Illkirch, France) following the manufacturer's instructions. For each well (e.g., 6 wells), add 10 μl of mimic/inhibitor (20 μM) to JetPRIME buffer, mix well, and vortex for 10 s after adding JetPRIME. Incubate at room temperature for 10 min to form the transfection complex. Add the mixture to 2 ml of cell culture medium to achieve a final concentration of 100 nM. Gently swirl the plate to homogenize.

### In vitro phagocytosis assays

2 × 10^5^ macrophages were seeded in a 6-well culture plate for 12 h. AT-Exos were washed and labeled with Red PKH26 membrane dye (Sigma-Aldrich, PKH26GL). After incubating macrophages in serum-free medium for 2 h, 1 × 10^12^ PKH26-labeled AT-Exos were added to the macrophages and subsequently kept at 37 °C for 2 h. Cells were washed with PBS, fixed with 2% paraformaldehyde and incubated with FITC-conjugated monoclonal antibody F4/80 (eBioscience, 11–4801-82) and 4',6-diamidino-2-phenylindole dihydrochloride (DAPI, eBioscience, D1306) sequentially. The cells were then imaged by an SP8 confocal microscope (Leica). The phagocytosis efficiency was determined as the PKH26-derived red fluorescence intensity per macrophage. The experiments were repeated three times, and for each individual experiment, 50 cells were imaged at random locations. All the settings for imaging and processing were kept constant, and the relative fluorescent intensities were calculated.

### Flow cytometry analysis

For surface marker staining, cells in the pellet were washed with PBS containing 0.5% BSA, incubated with Fc block (BD, 553141) and stained with LIVE/DEAD™ Fixable Aqua Dead Cell Stain Kit (Invitrogen, L34957), CD45-BV605 (BioLegend, 103140), CD3-PerCP (BioLegend, 100325), CD11b-PE (eBioscience, 12–0112-82), Ly6G-PE-cy7 (BioLegend, 127618), F4/80-FITC (eBioscience, 11–4801-82), CD206-Pacific Blue (BioLegend, 141717), iNOS-APC (eBioscience, 17–5920-82), Gr-1-AF700 (BioLegend, 108422), CD115-PerCP-cy5.5 (BioLegend, 135526), and F4/80-APC-cy7 (BioLegend, 123118) for 30 min in the dark. For detection of CD47 expression in AT-Exos, purified AT-Exos were labeled with Red PKH26 membrane dye (Sigma-Aldrich, PKH26GL) or PE-conjugated anti-CD63 antibody (Abcam, ab213090) according to the manufacturer’s guidelines. After centrifugation and resuspension with PBS, AT-Exos were stained with CD47-APC (BioLegend, 127514) for 30 min in the dark. Data acquisition was performed on an LSR Fortessa instrument (BD Biosciences) and analyzed by using FlowJo software (Treestar) and GraphPad Prism 6.0.

### Histological analysis

For immunohistochemistry, the tissues were fixed overnight in 10% formalin, embedded in paraffin and cut into 5-μm sections. Endogenous peroxidases were inactivated using 3% hydrogen peroxide. Nonspecific signals were blocked using 1% BSA. The samples were stained with the following primary antibody: UCP-1 (Sigma-Aldrich, U6382). After overnight incubation, the slides were washed and incubated with the secondary antibody (HRP-Polymer, Biocare Medical) for 30 min at room temperature. The slides were washed three times and stained with 3,3’-diaminobenzidine (DAB) substrate (Thermo Fisher Scientific, 34002). The slides were then counterstained with hematoxylin and mounted with mounting medium. In addition, hematoxylin–eosin (HE) staining was performed on paraffin-embedded tissue sections by the ST5010 Autostainer XL (Leica). Images were obtained with an Aperio Image Scope Viewer (Leica). Quantification was achieved by counting lipid droplets and UCP-1^+^ cell numbers in random areas for each sample.

### Immunoblot analysis

Whole-cell extracts were collected and lysed in RIPA lysis buffer (Beyotime Biotech, P0013) containing a cocktail of protease and phosphatase inhibitors (Beyotime Biotech, P1046). In addition, the exosome pellets were lysed with RIPA buffer. After centrifugation, separation by SDS-PAGE and transfer to PVDF membranes, immunoblotting was performed using specific primary antibodies, followed by a fluorescein-conjugated secondary antibody, and detected using an Odyssey fluorescence scanner (LI-COR). Primary antibodies against CD63 (Abcam, ab217345), p-mTOR (ab109268), mTOR (ab134903), SREBP-1 (ab313881), p-PI3K (ab278545), PI3K (ab133595), p-AKT (ab38449) and FASN (Abcam, ab128856) were purchased from Abcam. Antibodies against HSP70 (66183–1-Ig), CD9 (20597–1-AP), AKT (10176–2-AP) and β-actin (60008–1-Ig) were purchased from Proteintech. Antibodies against UCP-1 (U6382) were purchased from Sigma-Aldrich.

### Statistical analysis

For statistical comparisons, data were analyzed by unpaired Student’s t test or one-way ANOVA followed by Tukey’s post hoc multiple-comparison test, as appropriate. Statistical analysis was performed using Prism version 6.0 (GraphPad Software). Experimental values are presented as the mean ± SEM. *P < 0.05, **P < 0.01, ***P < 0.001 and ****P < 0.0001.

## Results

### Diet-induced obesity affects the lipid and miRNA composition of AT-Exos

**Fig. 1:**
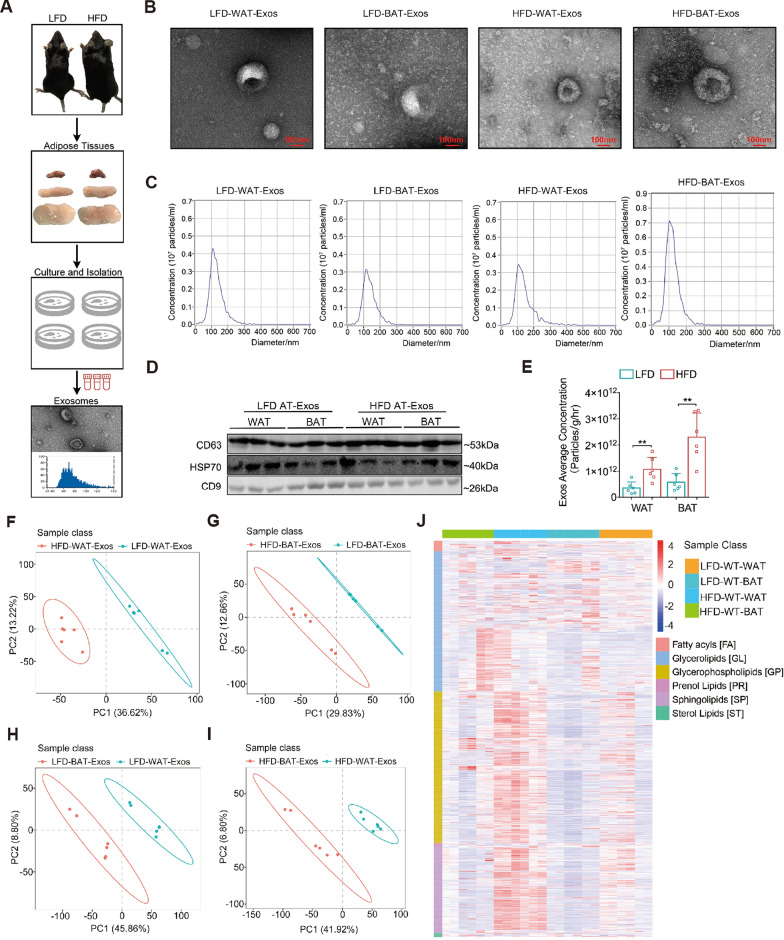
Diet-induced obesity affects lipid composition of AT-Exos. **A** Schematic diagram of AT-Exos isolation from HFD-fed or LFD-fed mice. **B** Representative electron microscopy images of exosomes. Scale bar, 100 nm. **C** The particle size of the AT-Exos measured by Nanoparticle tracking analysis. **D** The exosome-related protein markers CD63, HSP70 and CD9 measured by western blot in AT-Exos. These blots are representative of three independent replicate experiments. **E** Quantification of AT-Exos released from adipose tissue (per gram per hour) from HFD-fed and LFD-fed mice, as determined by Nanoparticle Tracking Analysis. n = 6 per group. **F-I** Partial Least-Squares Discriminant Analysis (PLS-DA) of lipid metabolites in AT-Exos from different origins, including WAT-Exos from HFD-fed and LFD-fed mice (panel f), BAT-Exos from HFD- and LFD-fed mice (panel g), BAT-Exos and WAT-Exos from LFD-fed mice (panel h), BAT-Exos and WAT-Exos from HFD-fed mice (panel i). **J** The heatmap shows relative content of different lipid types in WAT-Exos and BAT-Exos of wild-type B6 mice fed LFD or HFD for 16 weeks. n = 6 per group. All data are presented as mean ± SEM. *P < 0.05 and**P < 0.01

To describe the lipid and miRNA profiles of AT-Exos under diet-induced obesity, we isolated AT-Exos from age-matched wild-type C57BL/6 (B6) mice fed a high-fat diet (HFD) or low-fat diet (LFD) for 4 months (Fig. 1A). WAT-Exos (derived from vWAT) and BAT-Exos were collected, which were cup-shaped (Fig. 1B), ~ 100 nm in diameter (Fig. 1C) and positive for the exosomal markers CD63, HSP70 and CD9 (Fig. 1D). Recent studies have reported that obese vWAT released more exosomes than lean vWAT at the same time [23]. Consistent with this, we found the secretion of both WAT-Exos and BAT-Exos in the HFD group was much higher than that in the LFD group (Fig. 1E). In obesity, the lipidome profile is significantly altered. Recent studies have indicated that sphingolipids (SPs) and phosphatidylethanolamines (PE) are key drivers of cardiometabolic complications [31, 32]. We thus performed an untargeted lipidomics assay to evaluate the lipid composition in AT-Exos. Consequently, the lipid components of WAT-Exos from the HFD group markedly differed from those from the LFD group (Fig. 1F), characterized by an increase in sphingolipids and glycerophospholipids (GPs) in the HFD group, including PE, phosphatidylinositol (PI), phosphatidylserine (PS) and sphingomyelin (SM) (Table S3). Likewise, we observed a higher proportion of glycerol-lipids (GLs) and glycerophospholipids in BAT-Exos from the HFD group (Fig. 1G, J; Table S4). The exosomal composition is largely determined by its biogenesis and sources. Compared with BAT-Exos, WAT-Exos exhibited distinct lipid profiles under both LFD and HFD conditions (Fig. 1H, I). WAT-Exos appeared to contain less GLs and more GPs or SPs (Table S5, S6). Taken together, these data suggested that obesity-associated metabolism exert an impact on the biological process of AT-Exos, and the secretion pattern may have depot specificity.

**Fig. 2:**
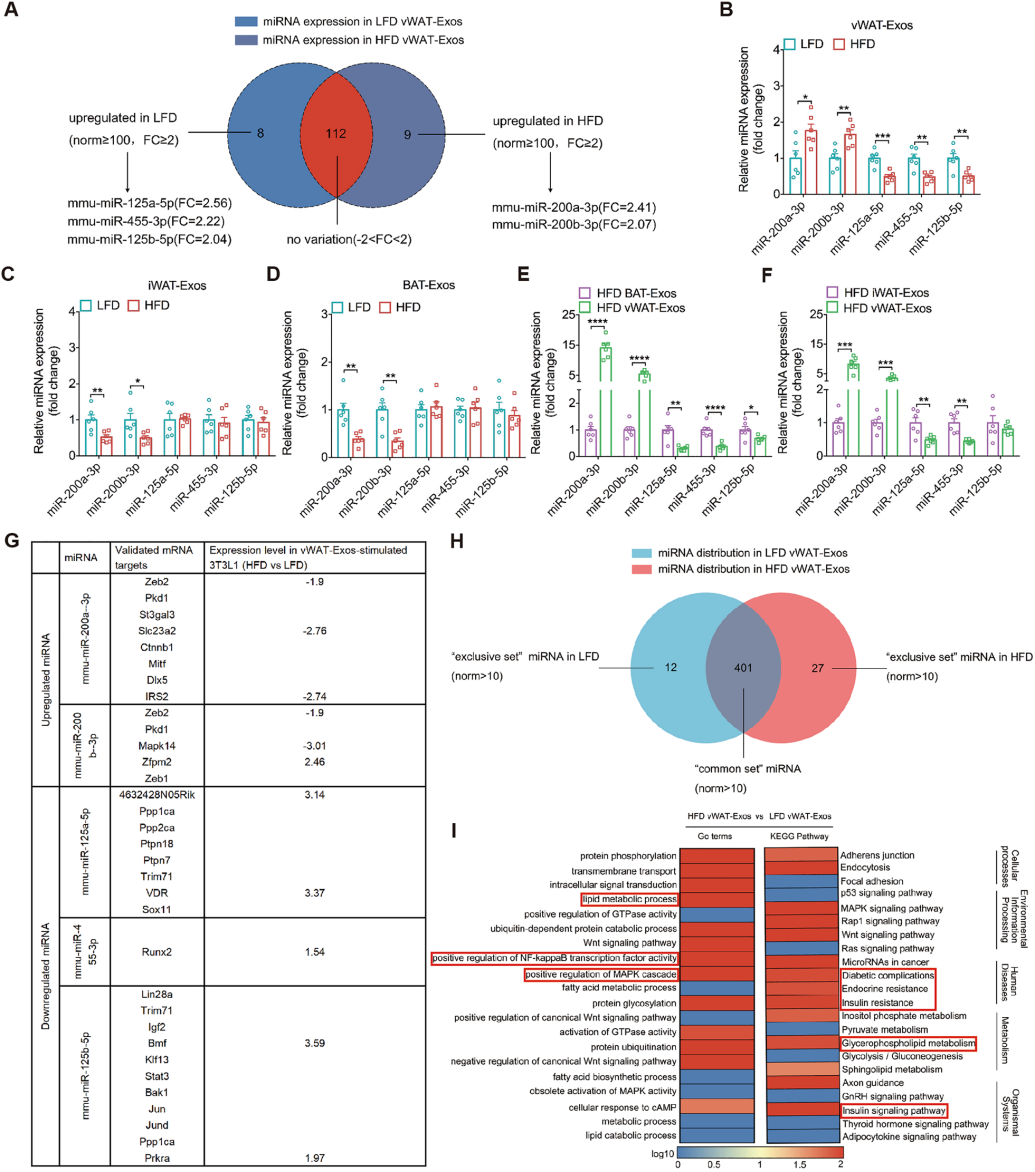
Diet-induced obesity affects miRNAs composition of AT-Exos. **A** Venn diagram between miRNA expression in vWAT-Exos using a fold change cutoff of 2 or greater (FC > 2), a value cutoff of P = 0.0001 and a norm value cutoff of 100. **B-D** qPCR validation of microarray data for miR-200a-3p, miR-200b-3p, miR-125a-5p, miR-455-3p and miR-125b-5p expression in vWAT-Exos (panel b), iWAT-Exos (panel c) and BAT-Exos (panel d) from HFD-fed mice relative to LFD-fed mice. Data are displayed as the expression of each miRNA in AT-Exos from HFD-fed mice relative to that assayed in LFD-fed mice, n = 6 per group. **E–F** qPCR validation of microarray data for miR-200a-3p, miR-200b-3p, miR-125a-5p, miR-455-3p and miR-125b-5p expression in vWAT-Exos relative to iWAT-Exos (panel e) or BAT-Exos (panel f) from HFD-fed mice. Data are displayed as the expression of each miRNA in vWAT-Exos from HFD-fed mice relative to that assayed in iWAT-Exos or BAT-Exos, n = 6 per group. **G** Validated mRNA target for each miRNA extracted from the miRTarBase are displayed as well as their respective expression level in 3T3L1 cells after stimulated by vWAT-Exos from HFD-fed mice compared with LFD-fed mice. **H** Venn diagram of the differential abundance of the identified miRNAs in vWAT-Exos from HFD-fed mice compared to LFD-fed mice using a norm value cutoff of 10. The number of “exclusive set” (red or blue area) and “common set” (gery area) of miRNAs is indicated on the diagram. **I** Based on the prediction of target genes for miRNA differences between vWAT-Exos from HFD-fed and those from LFD-fed mice, significantly enriched GO-terms/KEGG pathways were defined. The heatmap represents the log10 value of target genes number per GO term or per KEGG pathways. The red boxes highlight the significant GO-terms/KEGG pathways of interest. All data are presented as mean ± SEM. *P < 0.05, **P < 0.01, ***P < 0.001 and ****P < 0.0001

The role of miRNAs has been described in diabetes and obesity [33–35]. Although numerous studies reported a differential expression of miRNAs in adipose tissue from obese mice vs lean mice [36], no study so far has investigated their expression in AT-Exos. Using stringent statistical parameters (p ≤ 10^–4^, norm value ≥ 100, fold change [FC] ≥ 2), 8 miRNAs were identified as significantly downregulated and 9 miRNAs as significantly upregulated miRNAs in vWAT-Exos from HFD-fed mice comparing with those from LFD-fed mice (Fig. 2A). Based on the fold change values, the most strongly dysregulated miRNAs were selected for qPCR validations and further analyses. qPCR assays confirmed that miR-125a-5p, miR-455-3p and miR-125b-5p were downregulated in vWAT-Exos from the HFD group compared to those from the LFD group. Meanwhile, miR-200a-3p and miR-200b-3p levels were elevated (Fig. 2B). Based on previous researches [37–43], we identified miR-200a-3p and miR-200b-3p as “pro-obesity” factors, while miR-125a-5p, miR-125b-5p and miR-455-3p were “anti-obesity” factors. Given that different fat depots have unique characteristics and functions, and may play distinct roles in an obesogenic environment, we then investigated whether those miRNAs expression were also affected in iWAT-Exos and BAT-Exos. Interestingly, the expression of “anti-obesity” miRNAs did not significantly decrease in iWAT-Exos and BAT-Exos from obese mice comparing with their LFD-Exos (Fig. 2C, D). Unlike the tendency in vWAT-Exos, “pro-obesity” miRNAs were markedly downregulated in iWAT-Exos (Fig. 2C) or BAT-Exos (Fig. 2D) from the HFD group compared to the LFD group. Furthermore, the "pro-obesity" miRNAs in vWAT-Exos were notably higher than those in BAT-Exos (Fig. 2E) or iWAT-Exos (Fig. 2F), while the "anti-obesity" miRNAs were the opposite under HFD conditions. These findings suggested that the dysregulated expression of miR-200a-3p and miR-200b-3p occurs in a depot-selective manner and miRNA expression dysregulation in iWAT-Exos and BAT-Exos appeared to exhibit the similar patterns.

Considering that miRNAs can induce the degradation of their target mRNAs through 3'-untranslated region pairing, we next focused on experimentally validated mRNA targets (VTmRs) for each selected miRNA. Initially, mouse VTmRs were extracted from the miRTarBase [44] for each miRNA of interest (Fig. 2G). There were 8, 5, 8 and 11 VTmRs for miR-200a-3p, miR-200b-3p, miR-125a-5p and miR-125b-5p, respectively, whereas only one VTmR were extracted for miR-455-3p. This list of truly miRNA-sensitive RNAs was matched against their relative expression level in vWAT-Exos-stimulated mature 3T3-L1 adipocytes (referred to as 3T3-L1) when HFD group compared to LFD group (Fig. 2G). For miR-200a-3p, three VTmRs were indeed found to be downregulated above the 1.5-fold change threshold, consistent with the increased expression of miR-200a-3p in obese vWAT-Exos. These targets were zinc finger E-box binding homeobox 2 (*Zeb2*; FC: 1.9), Solute carrier family 23 member 2 (*Slc23a2*; FC: 2.76) and insulin receptor substrate 2 (*IRS2*; FC: 2.74). Similarly, the decreased expression of miR-200b-3p in obese vWAT-Exos matched with an increased expression of *Zeb2* (FC: 2.74) and Mitogen-activated protein kinase 14 (*Mapk14*; FC: 3.01) in 3T3-L1 (Fig. 2G). Among miR-125a-5p VTmRs, the expression of *4632428N05Rik* (FC: 3.14) and vitamin D receptor (*VDR*; (FC: 3.37) were upregulated, as expected from the miR-125a-5p decreased expression in obese vWAT-Exos (Fig. 2G). Notably, the expression of one miR-455-3p and two miR-125b-5p VTmRs were also upregulated (Fig. 2G). Taken together, this targeted approach enabled the identification of truly dysregulated mRNA targets for the selected miRNAs.

We then processed the data set for the distribution of miRNAs in vWAT-Exos. The Venn diagram of vWAT-Exos components analysis (norm value > 10) illustrated the distribution differences of miRNAs between HFD and LFD conditions, including “common set” and “exclusive set” (Fig. 2H). Of these, 12 miRNAs were identified as “exclusive set” in LFD-vWAT-Exos and 27 miRNAs were identified as “exclusive set” in HFD-vWAT-Exos (Fig. S1A). The distribution of 401 miRNAs was left unchanged in two groups. We further conducted target gene prediction of those “exclusive set” and “common set” miRNAs. Interestingly, we found that the predicted mRNA targets (PTmR) of exclusive miRNAs both in HFD and LFD group were enriched in lipid metabolism. Most potential targets were related to the inflammatory pathways involved in metabolic disorder (Fig. S1B), such as nuclear factor-kappaB (NF-kappaB) signaling pathway and c-jun N-terminal kinase (JNK) signaling pathway [45].Next, we performed enrichment analysis on the PTmR of the “common set” miRNAs described above. This yielded a list of GO-term for upregulated gene enrichment in HFD group, mainly including lipid metabolic process, NF-kappaB signaling and mitogen-activated protein kinases (MAPKs) signaling pathways (Fig. 2I). KEGG pathway enrichment analysis revealed that a higher number of differentially expressed genes were enriched in glycolipid metabolism-related pathways, such as glycerophospholipid metabolism, insulin resistance and the insulin signaling pathway in HFD group (Fig. 2I).These results suggest that dietary conditions can influence the composition and functionality of miRNAs in AT-Exos, with a primary focus on lipid metabolism and inflammatory signaling pathways that are implicated in metabolic dysregulation.

### miR-200a-3p and miR-200b-3p promote lipid accumualtion in 3T3L1 cells partially via PI3K/AKT/mTOR pathway

**Fig. 3:**
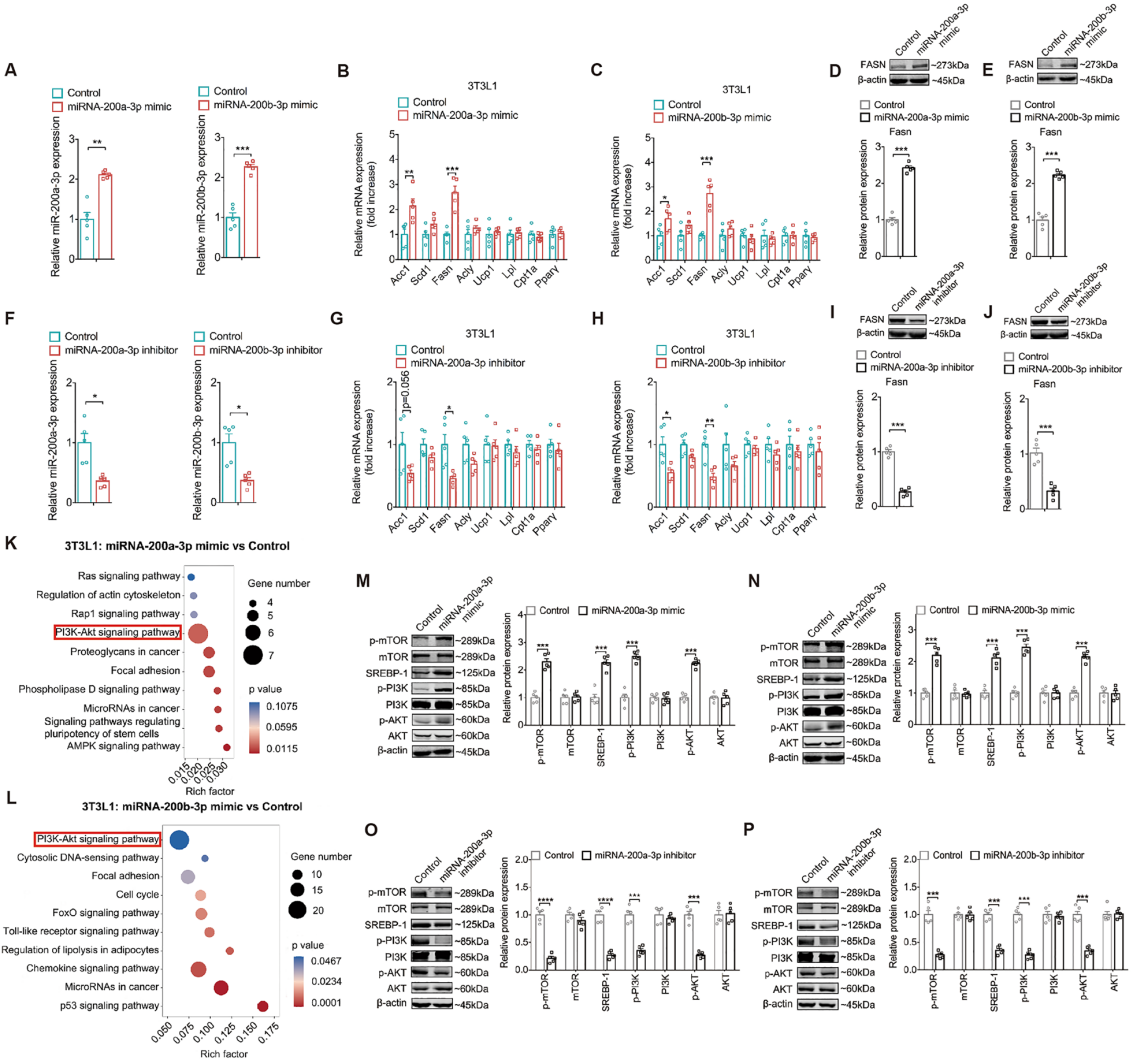
miR-200a-3p and miR-200b-3p promote the lipid accumulation in 3T3L1 cells partially via PI3K/AKT/mTOR pathway. **A** Normalized expression of miR-200a-3p and miR-200b-3p in 3T3L1 cells transfected with miR-200a-3p mimic and miR-200b-3p mimic. **B **and **C** Normalized expression of lipid metabolism-related genes in 3T3L1 transfected with miR-200a-3p mimic (panel b) and miR-200b-3p mimic (panel c). n = 5 per group. **D **and **E** Western blot analysis and quantification of FASN protein in 3T3L1 cells transfected with miR-200a-3p mimic (panel d) and miR-200b-3p mimics (panel e). **F** Normalized expression of miR-200a-3p and miR-200b-3p in 3T3L1 cells transfected with miR-200a-3p inhibitor and miR-200b-3p inhibitor. **G **and **H** Normalized expression of lipid metabolism-related genes in 3T3L1 transfected with miR-200a-3p inhibitor (panel g) and miR-200b-3p inhibitor (panel h). n = 5 per group. **I **and **J** Western blot analysis and quantification of FASN protein in 3T3L1 cells transfected with miR-200a-3p inhibitor (panel i) and miR-200b-3p inhibitor (panel j). **K **and **L** Top KEGG terms of the differentially expressed genes of 3T3L1 transfected with miR-200a-3p mimic (panel k) and miR-200b-3p mimic (panel l) based on RNA-seq analysis. **M **and **N** Western blot analysis and quantification of p-mTOR, mTOR, FASN, SREBP1, p-PI3K, PI3K, p-AKT and AKT protein in 3T3L1 transfected with miR-200a-3p mimic (panel m) and miR-200b-3p mimic (panel n). **O **and **P** Western blot analysis and quantification of p-mTOR, mTOR, SREBP1, p-PI3K, PI3K, p-AKT and AKT protein in 3T3L1 transfected with miR-200a-3p inhibitor (panel o) and miR-200b-3p inhibitor (panel p). All data are presented as mean ± SEM. *P < 0.05, **P < 0.01, ***P < 0.001 and ****P < 0.0001

miRNAs play significant roles in the pathophysiology of metabolic diseases. Several studies have revealed the putative roles of miR-200a-3p and miR-200b-3p in inflammation, insulin resistance, and lipid metabolism [37–39, 46]. To validate the effects of these “pro-obesity” miRNAs on mature 3T3-L1 adipocytes, miR-200a-3p and miR-200b-3p mimics were delivered into 3T3-L1 cells to induce overexpression. The transfection efficiency was confirmed by qPCR analysis (Fig. 3A). Next, we examined their effects on metabolic homeostasis. The results indicated that overexpression of miR-200a-3p or miR-200b-3p upregulated genes involved in fatty acid (FA) and triglyceride (TG) synthesis (fatty acid synthase [*Fasn*] and acetyl-CoA carboxylase 1 [*Acc1*]) (Fig. 3B, C). Consistently, the protein levels of FASN in 3T3-L1 cells were increased by miR-200a-3p or miR-200b-3p upregulation (Fig. 3D, E). We then validated the effects of miR-200a-3p and miR-200b-3p silencing on 3T3-L1 cell metabolism. Firstly, compared with the control group, miR-200a-3p and miR-200b-3p expression was significantly reduced in 3T3-L1 cells after treatment with their respective inhibitors (Fig. 3F). qPCR analysis further confirmed that silencing miR-200a-3p or miR-200b-3p inhibited lipid accumulation in 3T3-L1 cells (Fig. 3G, H). Western blot analysis revealed that FASN protein levels were decreased by miR-200a-3p or miR-200b-3p downregulation in 3T3-L1 cells (Fig. 3I, J). These results suggest that both miR-200a-3p and miR-200b-3p promote lipid accumulation in 3T3-L1 adipocytes.

Next, we investigated the underlying mechanism by which miR-200a-3p and miR-200b-3p activate obesity-related pathways using RNA-Seq. The data revealed significant upregulated enrichment of the phosphatidylinositol 3-kinase (PI3K)/protein kinase B (AKT) pathway in the miR-200a-3p and miR-200b-3p overexpression groups compared to the control group (Fig. 3K, L). As previously described, the PI3K/AKT/mammalian target of rapamycin (mTOR) pathway is closely associated with obesity and plays a crucial role in lipid accumulation [47]. A key downstream effector of AKT in lipid metabolism is sterol regulatory element-binding protein 1c (SREBP-1c) [48]. Adipocytes, in their role of energy storage, can reconstruct and accumulate dietary fats and synthesize triglycerides from non-lipid sources through a process known as de novo lipogenesis (DNL). Fatty acid synthase (FASN) and acetyl-CoA carboxylase 1 (ACC1) are essential for DNL and are highly expressed in adipose tissue under the control of SREBP-1c [49]. Therefore, we next examined whether miR-200a-3p and miR-200b-3p are involved in regulating the PI3K/AKT/mTOR signaling pathway. We overexpressed and downregulated miR-200a-3p or miR-200b-3p, respectively, and then detected changes in PI3K/AKT/mTOR-related proteins via Western blot. The results showed that the expression of phosphorylated PI3K (p-PI3K), phosphorylated Akt (p-AKT), phosphorylated mTOR (p-mTOR), and SREBP-1 was increased when miR-200a-3p or miR-200b-3p was upregulated (Fig. 3M, N), whereas downregulation of miR-200a-3p or miR-200b-3p had the opposite effects on these proteins in 3T3-L1 cells (Fig. 3O, P). Additionally, the total protein levels of PI3K, AKT, and mTOR were unchanged by either overexpression or downregulation of these “pro-obesity” miRNAs. In summary, these data indicated that miR-200a-3p and miR-200b-3p play function in 3T3-L1 cells through activation of the PI3K/AKT/mTOR pathway.

### AT-Exos affect inflammation and metabolic homeostasis in adipocytes

To further verify the effects of AT-Exos on metabolism in adipocytes, we isolated iWAT-Exos, vWAT-Exos and BAT-Exos from HFD-fed or LFD-fed mice and stimulated mature 3T3-L1 adipocytes. The transcription profiles of 3T3-L1 were examined by RNA-seq and confirmed by qPCR. We firstly analyzed the cell status of 3T3-L1 receiving LFD-Exos with its basal conditions. The data revealed that treatment with LFD-vWAT-Exos significantly reduced the production of cytokines, including IL-6 and TNF-α, in 3T3-L1 cells when compared to their levels in the baseline state (Fig. S2A, B). In contrast, LFD-iWAT-Exos (Fig. S2D, E) and LFD-BAT-Exos (Fig. S2G, H) were more likely to act as proinflammatory factors in 3T3-L1 cells, characterized by an enhanced inflammatory response and increased proinflammatory cytokines. Additionally, AT-Exos from LFD-fed mice promoted glycolysis and the glycogen synthesis, together with significantly attenuated cholesterol biosynthesis in 3T3-L1 cells, irrespective of their tissue origin (Fig. S2C, F, I). AT-Exos are rich in enzymes related to lipogenesis, such as ACC1 and FASN, which may affect lipogenic activity in target cells [16]. Under LFD-vWAT-Exos treatment, both the lipid synthesis and catabolic pathways were significantly suppressed in 3T3-L1 (Fig. S2C). Conversely, adipocytes showed enhanced lipid catabolism in the iWAT-Exos or BAT-Exos treatment groups, with upregulation of lipoprotein lipase (*Lpl*) and uncoupling protein 1 (*Ucp1*) expression (Fig. S2F, I).

We then assessed the role of HFD-Exos treatment in 3T3-L1 cells. Contrary to the effects of LFD-vWAT-Exos, treatment with HFD-vWAT-Exos caused an increase in proinflammatory cytokine levels (Fig. S3A, B). A similar inflammatory pattern was observed following stimulation with HFD-iWAT-Exos (Fig. S3D, E) or HFD-BAT-Exos (Fig. S3G, H), manifested as upregulation of the IL-1β, IL-6 and TNF-α. For the metabolic regulation, HFD-vWAT-Exos stimulation was found to promote lipogenesis in adipocytes (Fig. S3C). In parallel, HFD-iWAT-Exos and HFD-BAT-Exos exhibited similar effects on remodeling 3T3-L1 cells into a lipolytic state (Fig. S3F, I), which aligns roughly with the protective role of SAT on metabolic complications [50]. However, we failed to observe the remarkable changes in glucose metabolism after obese AT-Exos treatment.

**Fig. 4:**
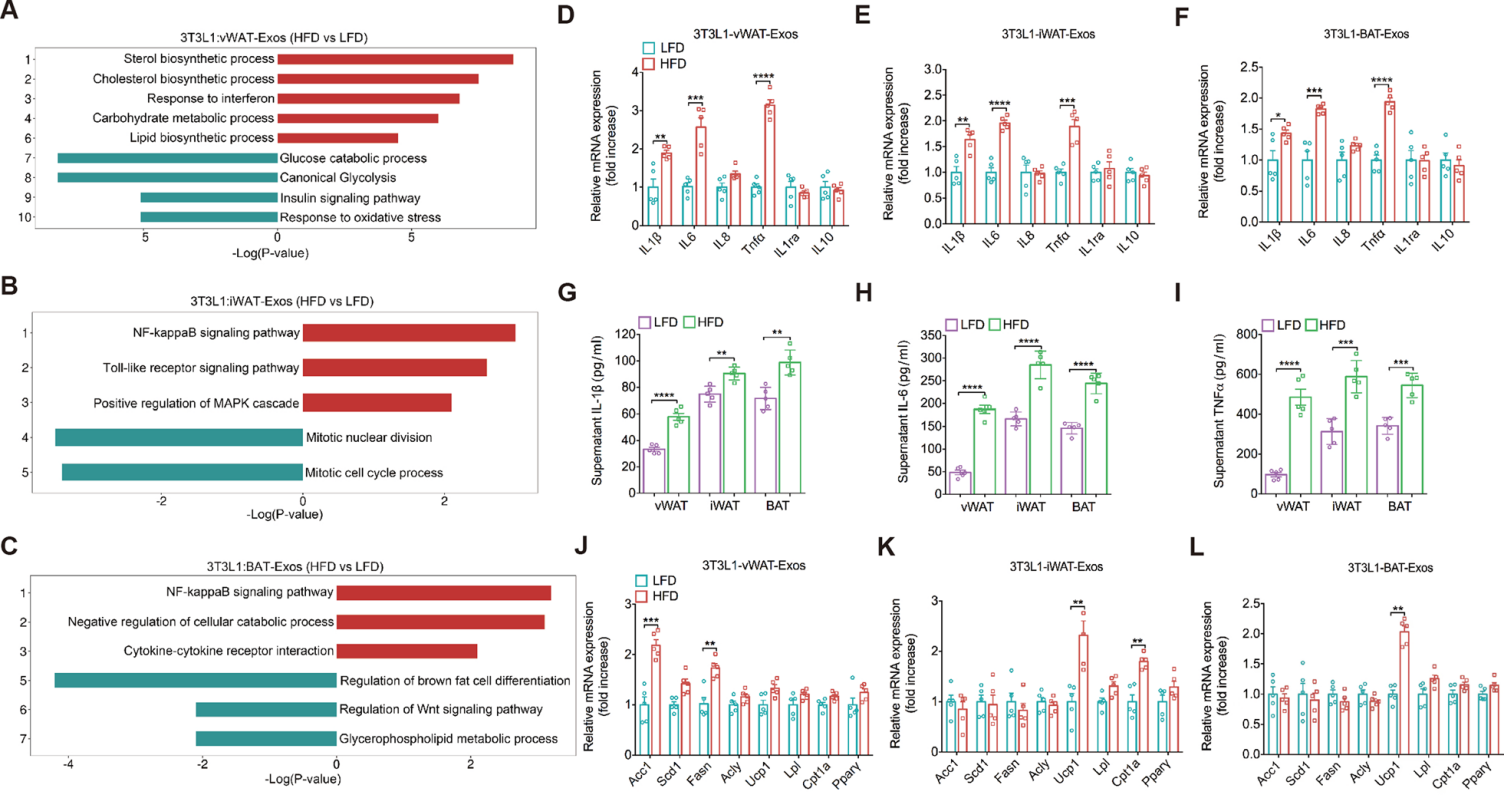
AT-Exos from different depots participate in inflammation and metabolic homeostasis of adipocytes. **A-C** Top GO terms of the differentially expressed genes of 3T3L1 treated with vWAT-Exos (panel a), iWAT-Exos (panel b) and BAT-Exos (panel c) derived from HFD-fed and LFD-fed mice based on RNA-seq analysis. Red box and blue box represent upregulated and downregulated GO terms, respectively. **D-F** Normalized expression of inflammation-related genes in 3T3L1 treated with vWAT-Exos (panel d), iWAT-Exos (panel e) and BAT-Exos (panel f) derived from HFD-fed and LFD-fed mice. n = 5 per group. **G-I** ELISA analysis of IL-1β (panel g), IL-6 (panel h) and TNF-ɑ (panel i) concentration of 3T3L1 supernatant treated with AT-Exos derived from HFD-fed and LFD-fed mice. n = 5 per group. **J-L** Normalized expression of lipid metabolism genes in 3T3L1 treated with vWAT-Exos (panel j), iWAT-Exos (panel k) and BAT-Exos (panel l) derived from HFD-fed and LFD-fed mice. n = 5 per group. All data are presented as mean ± SEM. *P < 0.05, **P < 0.01, ***P < 0.001 and ****P < 0.0001

Next, we assessed how obese AT-Exos from different depots, compared to lean AT-Exos, affected 3T3-L1 cells. Inflammation in adipose tissue is the main cause of obesity-related metabolic complications. Our data showed that pro-inflammatory pathways were significantly upregulated enrichment in obese AT-Exos treatment group compared to lean AT-Exos (Fig. 4A–C), as evidenced by various upregulated proinflammatory cytokines (Fig. 4D–F). Consistently, obese AT-Exos significantly increased the levels of IL-1β, IL-6, and TNF-α in the supernatant of cultured 3T3-L1 cells. (Fig. 4G–I). In terms of lipid metabolism, we observed that the lipid synthesis pathway was enriched in obese vWAT-Exo-treated cells (Fig. 4A). The genes involved in fatty acid and triglyceride synthesis *Fasn* and *Acc1* were highly upregulated, while the genes for adaptive thermogenesis (*Ucp1*, peroxisome proliferator-activated receptor γ [*Pparγ*], and carnitine palmitoyl-transferase 1a [*Cpt1a*]) were comparable to the expression in lean vWAT-Exos (Fig. 4J). Moreover, similar gene expression patterns were found between adipocytes incubated with obese iWAT-Exos and obese BAT-Exos compared to their lean counterparts **(**Fig. 4K, L). Immunoblotting of 3T3-L1 cells treated with AT-Exos confirmed the qPCR results (Fig. S4A). Notably, both vWAT-Exos and iWAT-Exos significantly suppressed glycolysis and glycogen synthesis in adipocytes under HFD conditions compared to LFD (Fig. S4B-D). Collectively, these findings indicated that AT-Exos play a role in modulating inflammation and glycolipid metabolism in adipocytes, with obese vWAT-Exos being particularly influential in promoting inflammation and lipid accumulation in diet-induced obesity.

To further understand the varied roles of AT-Exos in inflammation and metabolic homeostasis, we compared the impact of vWAT-Exos and iWAT-Exos on 3T3-L1 cells. Both lean (Fig. S4E, F) and obese iWAT-Exos (Fig. S4H, I) treatment triggered a stronger inflammatory response than vWAT-Exos. We also observed that vWAT-Exos and iWAT-Exos differed in the regulation of cellular glycolipid metabolic process in lean states. Lean vWAT-Exos treatment led to reduced cholesterol synthesis and fat metabolism but increased glycolysis and glycogen synthesis compared to lean iWAT-Exos (Fig. S4G). It was noteworthy that obese vWAT-Exos decreased lipolysis, including glycerol-lipid and triglyceride catabolic processes, more than obese iWAT-Exos (Fig. S4J-L). Despite both originating from "energy-storing" WAT, iWAT-Exos and vWAT-Exos have distinct biological effects, with iWAT-Exos taking on a "BAT-Exo-like" or "energy-expending" phenotype under diet-induced obesity conditions. We also collected exosomes from the liver and muscle tissues of mice fed either a LFD or HFD, termed liver-Exos and muscle-Exos (Fig. S5A-C), and used them to stimulate 3T3-L1 adipocytes. The results showed no significant differences in the regulation of adipocyte inflammation and lipid metabolism by liver-Exos or muscle-Exos from HFD-fed versus LFD-fed mice (Fig. S5D). Adipocyte biology is largely understood through cell culture models, especially 3T3-L1 cells. However, the 3T3-L1 model has limitations, such as reduced adipogenic potential over time and a 2-week requirement to form adipocytes. Stromal vascular fraction (SVF) from adipose tissue includes diverse cell types like mesenchymal stem cells, preadipocytes, endothelial cells, and M2 macrophages. These cells can differentiate into adipocytes due to their multipotent nature [51]. Additionally, OP9 cells, derived from mouse bone marrow stromal cells, can also undergo adipogenesis [52]. Therefore, we used the AT-Exos to stimulate both adipocyte models and obtained results similar to those seen in 3T3-L1 cells (Fig. S6, S7). Overall, our findings suggested that AT-Exos from different fat depots exert unique influences on adipocyte metabolism.

### Blocking the SIRPα-CD47 axis enhances macrophage phagocytosis of obese vWAT-Exos

**Fig. 5:**
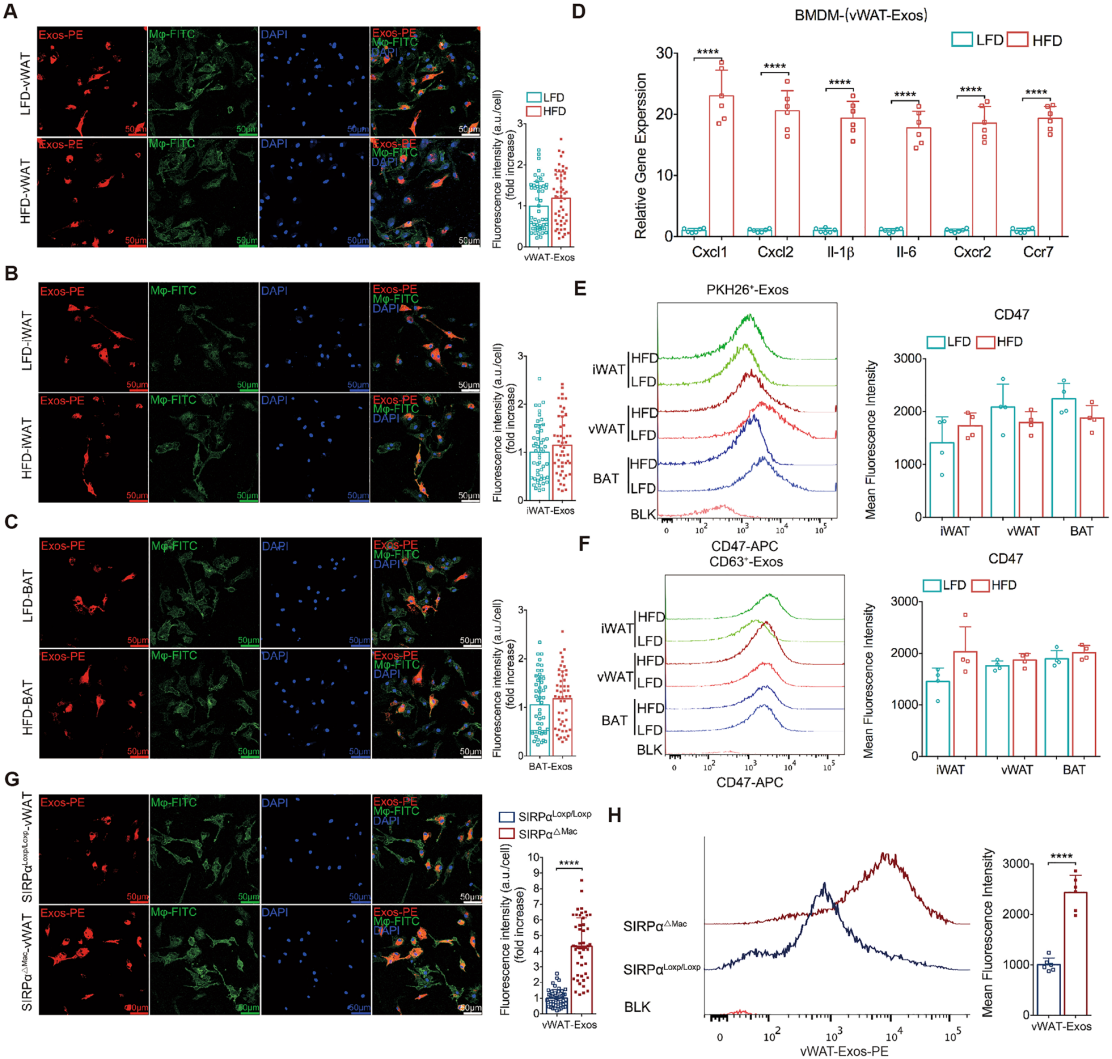
Blocking the SIRPα-CD47 axis enhances macrophage phagocytosis of obese vWAT-Exos. **A-C** Representative confocal microscopy images showing BMDMs (F4/80^+^ cells, green) phagocytosis of PKH26-labeled vWAT-Exos (panel a, left), iWAT-Exos (panel b, left) and BAT-Exos (panel c, left) derived from HFD-fed and LFD-fed mice, with quantitation shown in right. The cells nuclei were stained with DAPI (blue). n = 50 cells per group. Scale bar, 50 μm. **D** Normalized expression of inflammatory cytokine and chemokine genes in BMDMs treated with vWAT-Exos derived from HFD-fed and LFD-fed mice. n = 6 per group. **E **and **F** Flow cytometry analysis and quantitation of CD47 expression in PKH26^+^ AT-Exos (panel e) and CD63^+^ AT-Exos (panel f) derived from HFD-fed and LFD-fed mice. n = 4 per group. **G** Representative confocal microscopy images showing macrophage (F4/80^+^ cells, green) derived from HFD-fed SIRPα^ΔMac^ and SIRPα^loxp/loxp^ mice phagocytosis for PKH26-labeled vWAT-Exos (left), with quantitation shown in right. The cells nuclei were stained with DAPI (blue). n = 50 cells per group. Scale bar, 50 μm. **H** Flow cytometry analysis (left) and quantitation (right) of macrophage (F4/80^+^ cells) derived from HFD-fed SIRPα^ΔMac^ and SIRPα^loxp/loxp^ mice phagocytosis of PKH26.^+^ vWAT-Exos. n = 6 mice per group. All data are presented as mean ± SEM. *P < 0.05, **P < 0.01, ***P < 0.001 and ****P < 0.0001

To clarify how AT-Exos might link adipose tissue with macrophages, we performed phagocytosis experiments with bone marrow-derived macrophages (BMDMs) in vitro and found that the phagocytic-mediated clearance ability of obese or lean AT-Exos was comparable (Fig. 5A–C). Moreover, obese vWAT-Exos enhanced BMDM activation and migration more than lean vWAT-Exos, as evidenced by increased levels of proinflammatory (*Il-1β* and *Il-6*) and migration-related (C-X-C motif chemokine ligand 1 [*Cxcl1*], C-X-C motif chemokine ligand 2 [*Cxcl2*], C-X-C motif chemokine receptor 2 [*Cxcr2*], and C–C motif chemokine receptor 7 [*Ccr7*]) (Fig. 5D). Similar outcomes were observed with iWAT-Exos and BAT-Exos (Fig. S8A, B).

It is well established that the CD47-SIRPα pathway restrains macrophage phagocytosis by releasing a "don't eat me" signal [25]. Here, we examined CD47 levels in AT-Exos from HFD-fed mice versus LFD-fed mice. Flow cytometry revealed high CD47 expression on exosome surfaces, with minimal impact from diet-induced obesity (Fig. 5E, F). Considering the role of AT-Exos in adipose-inflammatory cell communication, we explored whether their removal could alleviate inflammation and metabolic disorders in obesity. Based on the expression of CD47 on exosomes, we hypothesized that blocking the recognition of CD47 and SIRPα on ATMs would enhance macrophage phagocytosis and clearance of AT-Exos. To test this, the Cre/loxP system was used to generate SIRPα^ΔMac^ (SIRPα^loxp/loxp^·Lyz-cre) mice, in which *sirpα* is deleted exclusively in macrophages (Fig. S8C). In vitro phagocytosis assays using immunofluorescence indicated that BMDMs from SIRPα^ΔMac^ mice engulfed more vWAT-Exos than those from SIRPα^loxp/loxp^ mice (Fig. 5G), with no notable difference in iWAT-Exos or BAT-Exos phagocytosis (Fig. S8D, E). Flow cytometry assays with BMDMs cocultured with labeled AT-Exos confirmed these findings (Fig. 5H). Collectively, these studies suggested that the SIRPα-CD47 axis plays a role in macrophage phagocytosis of obese vWAT-Exos.

### Blocking the SIRPα-CD47 axis attenuates vWAT-Exos-induced metabolic disorders

**Fig. 6:**
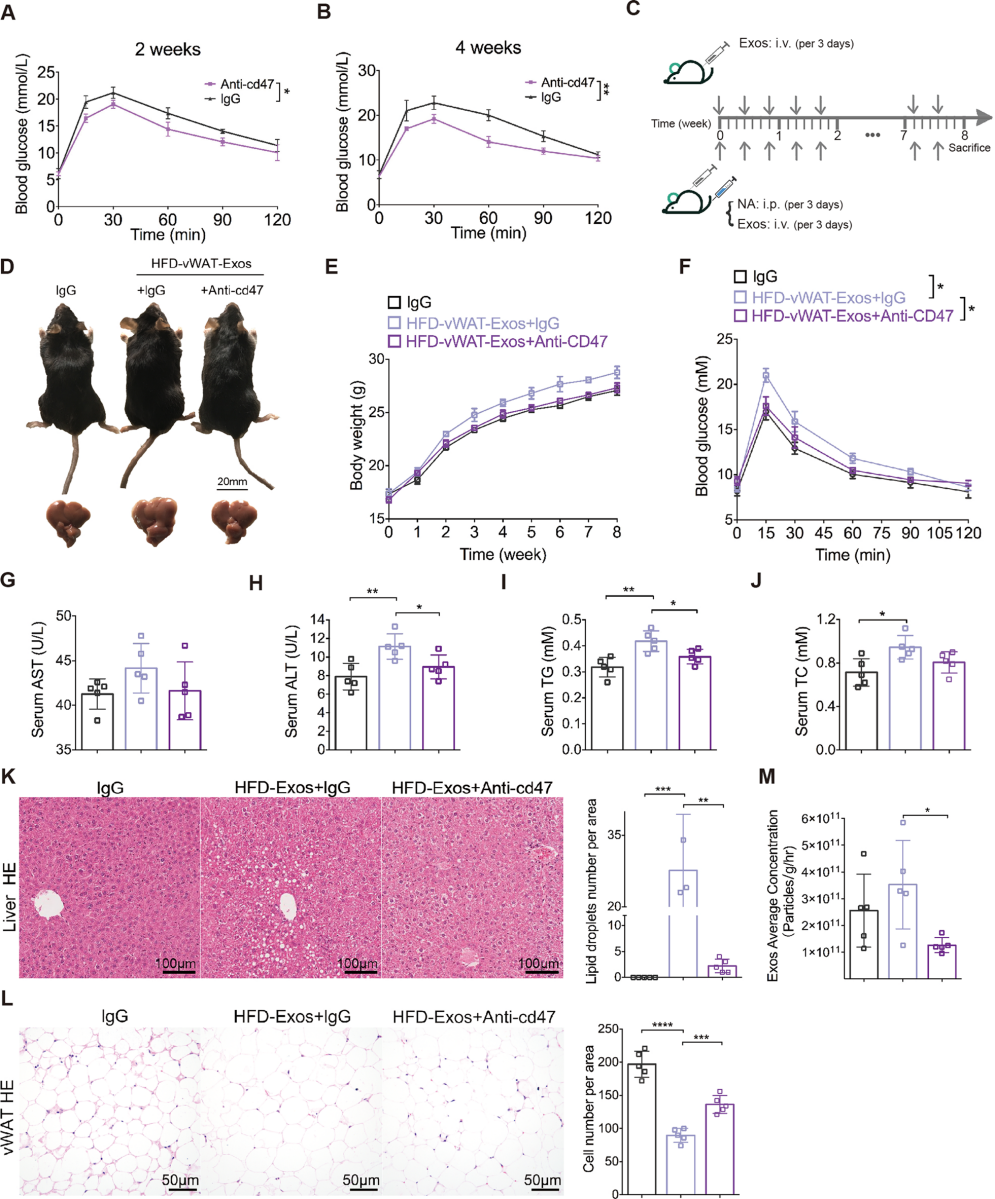
Blocking the SIRPα-CD47 axis attenuates vWAT-Exos-induced metabolic disorders. **A, B** Intraperitoneal glucose tolerance test (IPGTT, 2 g/kg) in mice injected with neutralizing antibodies targeting CD47 for 2 weeks (panel a) and 4 weeks (panel b). n = 5 mice per group. **C** Schedule diagram of mice treated with obese vWAT-Exos or in combination with neutralizing antibodies. **D, E** Representative images of mice (panel d, upper) and its liver (panel d, lower). Animals were injected with obese vWAT-Exos and neutralizing antibodies targeting CD47 for 8 weeks, with body weights were shown in (panel e). n = 5 mice per group. **F** IPGTT (2 g/kg) on those above mice. n = 5 mice per group. **G-J** Serum levels of AST (panel g), ALT (panel h), TG (panel i) and TC (panel j) in mice. n = 5 mice per group. **K** Representative images of HE staining of liver of mice treated with obese vWAT-Exos (left, n = 5 mice per group), and the quantification of lipid droplets number per area is shown (right). **L** Representative images of HE staining of vWAT sections of mice treated with obese vWAT-Exos (left), and the quantification of adipocytes number per area is shown in right. n = 5 mice per group. **M** Quantification of vWAT-Exos released by per gram per hour adipose tissue from mice treated with lgG, obese vWAT-Exos or obese vWAT-Exos combined with neutralizing antibodies targeting CD47. n = 5 per group. All data are presented as mean ± SEM. *P < 0.05, **P < 0.01, ***P < 0.001 and ****P < 0.0001

We next determined whether blocking SIRPα-CD47 with anti-CD47 antibodies affected metabolic homeostasis in mice. We injected neutralizing antibodies targeting CD47 intraperitoneally into B6 mice fed with a standard diet for 4 weeks starting at 2 months of age. Mice treated with NA-CD47 showed significant improvement in glucose tolerance at 2 and 4 weeks (Fig. 6A, B).

Given the association of obese vWAT-Exos with adipocyte inflammation and lipid metabolism, it is worthwhile to explore their impact on obesity and metabolic disorders in vivo. We injected obese vWAT-Exos intravenously into B6 mice every 3 days for 8 weeks starting at 2 months of age (Fig. 6C). As a result, the injection of obese vWAT-Exos led to glucose intolerance, with no significant body weight change (Fig. 6D–F). In addition, the treatment with obese vWAT-Exos resulted in elevated serum levels of ALT, TG and TC in mice (Fig. 6G–J). Hepatic steatosis was also worsened by exosome delivery, manifested as a great increase in macro-vesicular steatosis (Fig. 6K). Histological examination of adipocytes in the visceral fat pads using HE staining showed a marked increase in size (Fig. 6L). These data suggested that vWAT-Exos alone acted as critical mediators linking obesity and metabolic dysregulation.

To evaluate the potential of CD47 blockade to alleviate metabolic disturbances caused by obese vWAT-Exos, we delivered a neutralizing antibody via intraperitoneal injection to the exosome-treated mice (Fig. 6C). Although minor variations in body weight were observed **(**Fig. 6E**)**, the mice treated with NA-CD47 exhibited improved glucose tolerance (Fig. 6F) and a decrease in serum ALT and TG levels (Fig. 6H, I). Moreover, the administration of NA-CD47 attenuated hepatic steatosis and reduced adipocyte hypertrophy (Fig. 6K, L). Interestingly, we found that the secretion of vWAT-Exos in the NA-CD47-treated group was much lower than that in the control group (Fig. 6M). Therefore, it is evident that blocking the SIRPα-CD47 axis can effectively inhibit obese vWAT-Exo-induced metabolic disorders, partly due to the clearance effect of vWAT-Exos.

### SIRPα deficiency in macrophages protects mice from diet-induced obesity

**Fig. 7:**
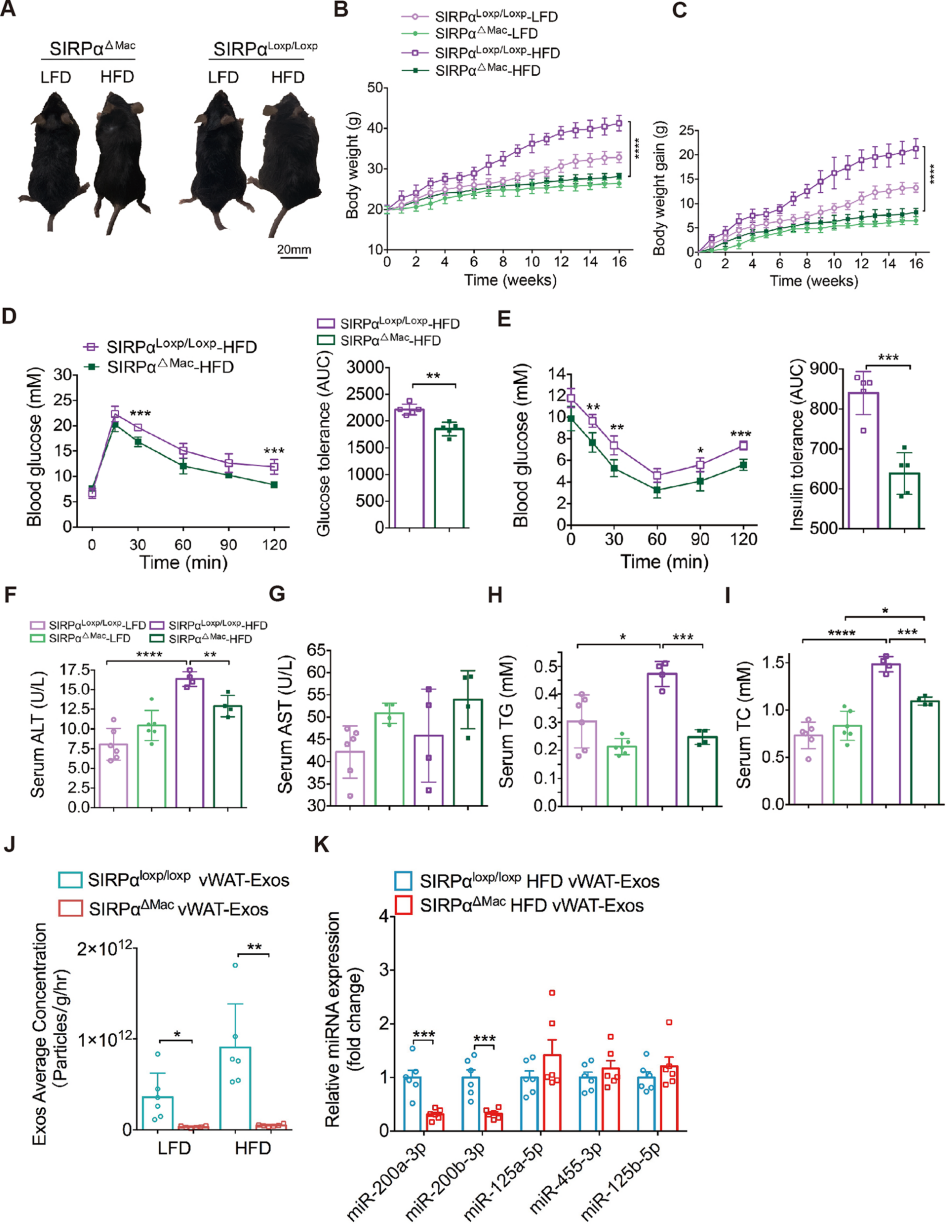
SIRPα deficiency in macrophages protects mice from diet-induced obesity. **A-C** A representative image (panel a), weekly body weight (panel b) and body weight gain per mouse (panel c) of SIRPα^ΔMac^ and SIRPα^loxp/loxp^ mice. Animals were fed with LFD or HFD for 16 weeks. n = 6–9 mice per group. **D** IPGTT (2 g/kg) on SIRPα^ΔMac^ and SIRPα^loxp/loxp^ mice fed with HFD for 16 weeks (left). Area under cure (AUC) is shown in right. n = 5 mice per group. **E** IPITT (0.75 mU/kg) on SIRPα^ΔMac^ and SIRPα^loxp/loxp^ mice fed with HFD for 16 weeks (left). Area under cure (AUC) is shown in right. n = 6 mice per group. **F-I** Serum levels of AST (panel f), ALT (panel g), TG (panel h) and TC (panel i) in mice. n = 4-6 mice per group. **J** Quantification of vWAT-Exos released by per gram per hour adipose tissue from SIRPα^ΔMac^ and SIRPα^loxp/loxp^ mice under HFD or LFD conditions. Absolute concentration of exosomes in culture supernatant of adipose tissues. n = 6 per group. **K** Normalized expression of miR-200a-3p, miR-200b-3p, miR-125a-5p, miR-455-3p and miR-125b-5p in vWAT-Exos from HFD-fed SIRPα^ΔMac^ mice compared to HFD-fed SIRPα.^loxp/loxp^ mice. n = 6 per group. All data are presented as mean ± SEM. *P < 0.05, **P < 0.01, ***P < 0.001 and ****P < 0.0001

To elucidate the effects of *Sirpα* deletion in macrophages, we assigned SIRPα^ΔMac^ mice and their SIRPα^loxp/loxp^ littermates to either a HFD or LFD for 16 weeks. Upon HFD treatment, SIRPα^ΔMac^ mice had a substantial attenuated body weight gain compared to SIRPα^loxp/loxp^ mice (Fig. 7A–C). HFD-induced elevation of serum insulin was greater in SIRPα^loxp/loxp^ mice than in SIRPα^ΔMac^ mice (Fig. S9A), suggesting exacerbated insulin resistance in SIRPα^loxp/loxp^ mice. Indeed, HFD-fed SIRPα^ΔMac^ mice had better glucose tolerance and insulin sensitivity (Fig. 7D, E). In keeping with the lower body weight in HFD-fed SIRPα^ΔMac^ mice, these animals showed reduced circulating levels of ALT and AST (Fig. 7F, G), TG and TC (Fig. 7H, I), as well as HDL-c and LDL-c (Fig. S9B, C) relative to the SIRPα^loxp/loxp^ mice.

We also found that SIRPα^ΔMac^ mice accumulated notably less fat mass than SIRPα^loxp/loxp^ mice, while lean mass was unchanged between the two HFD groups (Fig. S9D-F). In accordance with the decreased adiposity, the HFD-induced increase in serum leptin, whose expression correlates with adiposity [53], was lower in SIRPα^ΔMac^ mice than in SIRPα^loxp/loxp^ mice (Fig. S9G). The reduction in adipose tissue mass was not due to alterations in daily food intake (Fig. S9H), suggesting an increase in energy expenditure in SIRPα^ΔMac^ mice. Analyses of these animals revealed higher O_2_ consumption (VO_2_), CO_2_ production (VCO_2_) and locomotor activity during both the light and dark phases (Fig. S9I-K). We also found that the secretion of vWAT-Exos in the SIRPα^ΔMac^ mice was much lower than that in the SIRPα^loxp/loxp^ mice whether on the HFD or LFD conditions (Fig. 7J). Consistently, the expression of “pro-obesity” miRNAs were markedly downregulated in obese vWAT-Exos from SIRPα^ΔMac^ mice compared to SIRPα^loxp/loxp^ mice, while the “anti-obesity” miRNAs were not significantly different between the two groups (Fig. 7K). Therefore, the dysregulation of miRNA expression in SIRPα^ΔMac^ mice, at least partially, led by increased phagocytosis of obese vWAT-Exos by macrophages. Altogether, these results indicated that *Sirpα* deletion in macrophages caused improved glucose disposal, increased energy expenditure, reduced weight gain and decreased “pro-obesity” miRNAs in diet-induced obesity.

### SIRPα deficiency in mice alleviates HFD-induced lipid dysregulation and tissue inflammation

**Fig. 8:**
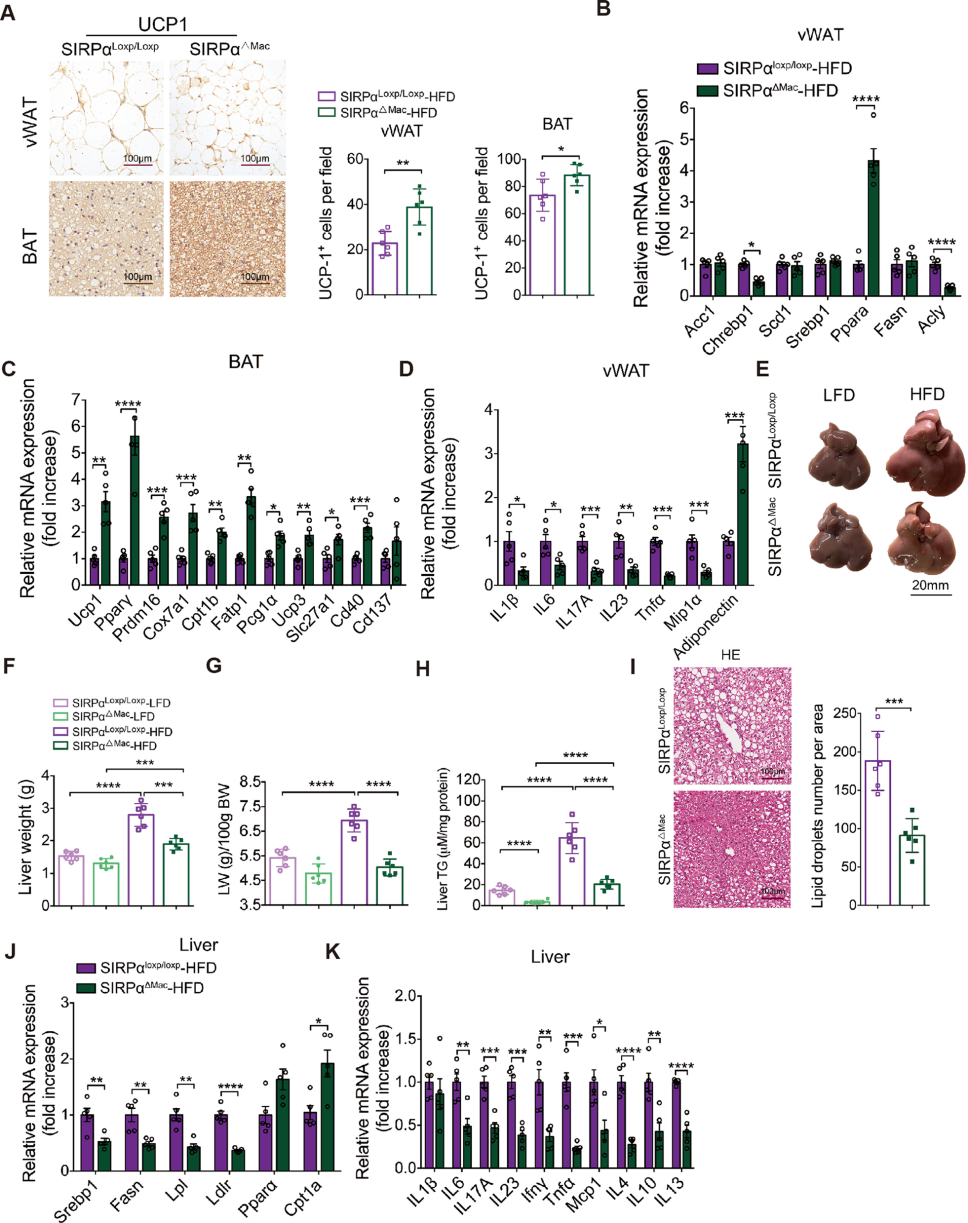
SIRPα deficiency in mice alleviates HFD-induced lipid dysregulation and tissue inflammation. **A** Representative image of UCP1 immunostaining in vWAT and BAT sections of SIRPα^ΔMac^ and SIRPα^loxp/loxp^ mice fed with HFD for 16 weeks (left), and the quantification of UCP1-positive area in vWAT (middle) and BAT (right) is shown. n = 6 per group. Scale bar, 100 μm. **B** Normalized expression of lipogenesis-related genes in vWAT of SIRPα^ΔMac^ and SIRPα^loxp/loxp^ mice fed with HFD for 16 weeks. n = 5 per group. **C** Normalized expression of mitochondrial oxidation and thermogenic genes in BAT of SIRPα^ΔMac^ and SIRPα^loxp/loxp^ mice fed with HFD for 16 weeks. n = 5 per group. **D** Normalized expression of genes including inflammatory cytokine, chemokine and adiponectin in vWAT of SIRPα^ΔMac^ and SIRPα^loxp/loxp^ mice fed with HFD for 16 weeks. n = 5 per group. **E** Representative images of liver of SIRPα^ΔMac^ and SIRPα^loxp/loxp^ mice fed with LFD or HFD for 16 weeks. **F** Absolute weight of liver was measured immediately following sacrifice. n = 6 mice per group. **G** The ratio of liver weight (LW) and body weight (BW) of each mouse. n = 6 mice per group. **H** Hepatic levels of TG in SIRPα^ΔMac^ and SIRPα^loxp/loxp^ mice fed with LFD or HFD for 16 weeks. n = 6 mice per group. **I** Representative images of HE staining of liver sections of SIRPα^ΔMac^ and SIRPα^loxp/loxp^ mice fed with HFD for 16 weeks (left), with quantification of lipid droplets number per area is shown in right. n = 6 mice per group. **J** Normalized expression of lipid synthesis and uptake genes in liver of SIRPα^ΔMac^ and SIRPα^loxp/loxp^ mice fed with LFD or HFD for 16 weeks. n = 5 per group. **K** Normalized expression of inflammatory cytokine genes in liver of SIRPα^ΔMac^ and SIRPα.^loxp/loxp^ mice fed with HFD for 16 weeks. n = 5 per group. All data are presented as mean ± SEM. *P < 0.05, **P < 0.01, ***P < 0.001 and ****P < 0.0001

Since blocking the SIRPα-CD47 axis eases lipid accumulation and inflammation from obese vWAT-Exos, we examined if the SIRPα^ΔMac^ mice exhibit similar phenotypes under HFD conditions. Histological analysis of vWAT and BAT revealed enhanced features of beiging WAT in SIRPα^ΔMac^ mice, such as multilocular lipid droplets and higher expression of UCP-1 (Fig. 8A). These animals also had higher expression of thermogenic genes in vWAT and BAT (Fig. 8B, C). The gene ATP citrate lyase (*Acly*), which encodes a key enzyme of de novo fatty acid synthesis, was highly downregulated in the vWAT of SIRPα^ΔMac^ mice (Fig. 8B). Previous study has reported that carbohydrate-responsive element-binding protein (*Chrebp*) emerged as a major mediator of glucose action on lipogenic gene expression and as a key determinant of lipid synthesis [54]. Here, we noted a significant reduction in the mRNA level of *Chrebp1* in SIRPα^ΔMac^ mice. In contrast, peroxisome proliferator-activated receptor-α (*Pparα*) was markedly increased in the vWAT of SIRPα^ΔMac^ mice (Fig. 8B). vWAT has been suggested to be the primary source of cytokine and adipokine release within obesity-associated inflammation [55]. Our data showed a decrease in proinflammatory cytokines in vWAT from SIRPα^ΔMac^ mice compared to SIRPα^loxp/loxp^ mice under HFD condition. In contrast, the level of adiponectin was drastically elevated in vWAT from HFD-SIRPα^ΔMac^ mice (Fig. 8D). Increased accumulation of adipose tissue macrophages is a significant contributor to obesity-induced chronic inflammation [19, 56]. To eliminate its possible impact on the inflammation level, we determined the amounts of ATMs in adipose tissues between SIRPα^ΔMac^ mice and SIRPα^loxp/loxp^ mice by flow cytometry (Fig. S10A). Interestingly, no obvious differences were observed in the adipose tissues. The patterns of neutrophil and T-cell levels in SIRPα^ΔMac^ mice were also similar to those in SIRPα^loxp/loxp^ mice (Fig. S10B-D). In addition, we found that the two groups of mice contained a similar ratio of M1 and M2 macrophages in adipose tissues, implying that *Sirpα* deficiency in macrophages was insufficient to affect their polarization (Fig. S10E). We further explored whether loss of *Sirpα* affects macrophages in the bone marrow (BM) and spleen. The data revealed that the relative frequency of these cells did not differ between SIRPα^ΔMac^ and SIRPα^loxp/loxp^ mice (Fig. S10F-H). Collectively, the SIRPα^ΔMac^ mice showed a favorable metabolic profile compared with the SIRPα^loxp/loxp^ mice under HFD conditions. The net effects of *Sirpα* deficiency were to reduce inflammation and lipid biosynthesis in vWAT and to activate BAT.

Liver and adipose tissue interact with hormones and other biologically active factors to jointly maintain the body’s homoeostasis [57]. We therefore explored whether *Sirpα* deletion had beneficial effects on lipid metabolism and inflammation in the liver. Under HFD conditions, the liver weight (Fig. 8E–G), hepatic triglyceride levels (Fig. 8H) and hepatic lipid deposition (Fig. 8I) were lower in SIRPα^ΔMac^ mice than in SIRPα^loxp/loxp^ mice. Consistently, the hepatic expression of genes for lipid synthesis and uptake, as well as proinflammatory factors, was decreased in HFD-fed SIRPα^ΔMac^ mice compared to SIRPα^loxp/loxp^ mice (Fig. 8J, K). In contrast, the expression of *Cpt1a*, which is associated with β-oxidation, was significantly upregulated (Fig. 8J). These results provided evidence that SIRPα deficiency in macrophages protected mice from hepato-steatosis when fed with HFD. Since visceral adiposity and hepatic steatosis are etiologically intertwined [58], the metabolic improvement in these tissues may reflect a secondary effect resulting from obesity resistance in SIRPα^ΔMac^ mice.

We further used genetically obese ob/ob mice to validate the strategy of blocking the SIRPa-CD47 axis in the treatment of obesity. Five-week-old male ob/ob mice were treated with IgG and NA-CD47 for 8 weeks (Fig. S11A). After 8 weeks of NA-CD47 treatment, ob/ob mice did not show significant weight loss (Fig. S11B). However, these mice exhibited improved glucose tolerance (Fig. S11C), reduced hepatic steatosis (Fig. S11D, E), and slightly decreased adipocyte hypertrophy (Fig. S11F). Moreover, NA-CD47 treatment only reduced serum TG but not AST, ALT, or TC levels (Fig. S11G-J). Consistent with the vWAT-Exos intraperitoneal injection model, NA-CD47 treated ob/ob mice had reduced secretion of vWAT-Exos compared to controls (Fig. S11K). These findings suggest that blocking the SIRPα-CD47 axis modestly alleviates metabolic disorders in ob/ob mice, partly by reducing vWAT-Exos carrying pro-lipid accumulation signals.

## Discussion

Adipose tissue is a multifunctional organ distributed across the body in numerous locations and is involved in the management of energy metabolism via the secretion of adipokines, hormones and exosomes. Exosomes are rich in proteins, lipids, and RNA molecules, all of which provide exosomes with a wide spectrum of potential effects on target cells [13]. In this study, we conducted lipidomics and miRNA sequencing on AT-Exos derived from HFD-fed and LFD-fed mice. We analyzed the lipid composition, miRNA expression, and potential functions of these miRNAs based on target genes prediction. Prior research has indicated that obese children have a lipid profile linked to liver and glucose metabolism issues, with increased ceramides and phospholipids and decreased sphingomyelins [59]. Our findings showed that AT-Exos lipid content correlates with dietary status and adipose tissue type. Notably, diet-induced obesity markedly elevated the SPs, PE, PI and PS levels in vWAT-Exos, underscoring their role in the condition.

Multiple studies have shown that exosome-associated RNAs modulate the physiological functions and pathological processes in metabolic disorders [60, 61]. In obesity, adipose tissue hypertrophy alters the miRNA profile of plasma exosomes and affects glucose uptake and lipid metabolism in mice [62]. miR-200a-3p, highly expressed in type 1 diabetes, targets several metabolic pathways, including fatty acid β-oxidation and the pentose phosphate pathway [37]. It was also implicated in PM2.5-induced glucose metabolism disruption and lipidome changes [63]. miR-200b-3p can exacerbate Atherosclerosis by promoting lipid accumulation and inhibiting cholesterol efflux in foam cells [38]. Moreover, it has been reported to be remarkably increased in NAFLD rat livers, affecting the cholesterol synthesis [39]. On the other hand, miR-125a-5p is involved in adipogenesis and may inversely correlate with HFD-induced obesity [40]. miR-125b-5p enhances insulin sensitivity and pancreatic β-cell function in type 2 diabetes [41]. It was reduced in NAFLD clinical samples and mice on a high cholesterol diet, and targeted to ITGA8 to inhibit NAFLD progression [42]. Additionally, miR-455 has been found to play a critical role in BAT adipogenesis [43]. In this study, the upregulation of miR-200a-3p and miR-200b-3p, along with the downregulation of miR-125a-5p, miR-125b-5p, and miR-455-3p in vWAT-Exos from obese mice, suggested these miRNAs' potential role in obesity-related metabolism. This study investigated the role and underlying mechanisms by which miR-200a-3p and miR-200b-3p regulated the development of obesity-associated inflammation and dysregulation of lipid metabolism, and we evidenced that miR-200a-3p and miR-200b-3p activated the PI3K/AKT/mTOR signaling pathway, resulting in the lipid accumulation and promoting obesity-associated metabolism disorder. miR-200a-3p and miR-200b-3p/PI3K/AKT/mTOR pathway may be an important mediator of obesity-associated metabolic homeostasis, and could serve as targeted therapy strategy for obesity. Over the past decades, the pathogenesis of obesity has been extensively investigated, and an increasing number of signal transduction pathways have been implicated in obesity [64]. Those pathways involved in adipogenesis, adipose tissue inflammation, insulin sensitivity and energy expenditure, such as Wnt signaling pathway [65–67], MAPK signaling pathway [68–70] and NF-kappaB signaling pathway [45, 71, 72]. We found that diet-induced obesity affects the multiple functions of miRNAs associated with adipose tissue metabolism and inflammation mentioned above. Therefore, studies on the internal components of AT-Exos will help us make better use of this endogenous delivery machine for various regulatory functions.

Given the heterogeneity of adipose tissue, depot-specific exosomes may play distinct roles in bodily homeostasis and systemic metabolism. An important aspect of our study is to elucidate how different types of AT-Exos influence adipocyte inflammation and metabolic balance. Interestingly, although both originate from white adipose tissue, we found that vWAT-Exos and iWAT-Exos exhibit diametrically opposed functions and effects on metabolism. Consistent with this, previous studies have showed that visceral adipocytes have enhanced lipolysis rate and susceptibility to cell apoptosis [73], while subcutaneous preadipocytes have enhanced lipid accumulation and proliferation rate [74]. Our data suggested that these disparities may be due to the modifications of distinct exosome-associated signals. However, the exact mechanism of functional diversification requires further exploration. Recent studies have recognized AT-Exos as a type of intercellular communication mediator involved in lipid metabolism [75], insulin resistance [76], inflammation and immunity. Our findings confirmed that obese vWAT-Exos significantly contribute to the development of obesity and metabolic dysregulation. Since our study was performed in rodents, additional studies on humans are necessary to determine the functions of vWAT-Exos in detail.

Previous studies have demonstrated that ATMs contribute to a proinflammatory state in obesity and obesity-associated metabolic dysfunctions [19, 77]. In the obese state, there is an increase in macrophage infiltration into adipose tissue, which often undergoes a phenotypic shift known as ATM polarization [78]. Various metabolic organs, such as adipose tissue, liver and skeletal muscle, interact with ATMs by secreting exosomes [79]. More recently, adipocyte-derived exosomes have been considered as a remarkable mediator in adipocyte/macrophage crosstalk. For instance, adipocyte-derived exosomes rich in miR-34a inhibit M2 polarization by inhibiting the expression of Krüppel-like factor 4 (*Klf4*) [80], whereas exosomes from adipose stem cells (ADSCs) are found to promote M2 polarization in macrophages [81]. In this study, we revealed that all three types of obese AT-Exos can promote macrophage activation and migration compared to lean AT-Exos in vitro. Interestingly, the phagocytic ability of macrophages towards AT-Exos is irrespective of dietary conditions. The underlying mechanism by which AT-Exos participate in adipocyte-macrophage crosstalk and modulate adipose tissue inflammation remains to be further explored.

It is well established that the SIRPα-CD47 axis acts on inhibitory phagocytic signals. In view of the high expression of CD47 on exosomes, we found that blockade of the SIRPα-CD47 axis enhances macrophage phagocytosis of vWAT-Exos, but not iWAT-Exos or BAT-Exos. It is worth paying attention to whether this selective phagocytosis was due to exosomes-associated differential signals. Moreover, administration of neutralizing antibodies targeting CD47 can alleviate the metabolic disorder in vivo caused by obese vWAT-Exos. Consistently, we observed that SIRPα deficiency in macrophages protects mice from diet-induced obesity. Nevertheless, loss of SIRPα had no impact on macrophage infiltration and polarization in the context of obesity.

Similar to obesity, cancer is a systemic, multisystem disease characterized by a wide range of pathophysiological changes, including systemic metabolic disorders, immune suppression, cardiovascular damage and more. Recent studies have highlighted the role of the SIRPα-CD47 axis in cancer immune suppression, revealing its complex dual roles in immune evasion and treatment. On one hand, CD47 overexpression allows cancer cells to evade immune clearance by binding to SIRPα. This mechanism is common in various cancers, associated with poor prognosis [82–84], and impacts tumor microenvironment [85],epithelial-mesenchymal transition (EMT) and cancer cell stemness [86]. On the other hand, CD47 overexpression offers a new immunotherapy target. Blocking the CD47-SIRPα pathway enhances macrophage phagocytosis and promotes adaptive immune responses [87]. Anti-CD47 monoclonal antibodies have shown promise in clinical trials, achieving partial remission in some patients. However, adverse effects like anemia limit their broad application [88]. Looking ahead, future research should focus on further elucidating the mechanisms of the SIRPα-CD47 axis across different tissues and pathological conditions. This will involve developing specific inhibitors targeting this axis and rigorously evaluating their safety and efficacy in clinical settings. Such efforts hold the potential to advance the treatment of not only cancer but also other systemic diseases like obesity, thereby offering new hope for patients affected by these complex and multifaceted conditions.

## Conclusion

We pioneered a systematic analysis of lipids and miRNAs composition of AT-Exos produced by visceral white and brown adipose tissue of HFD-fed or LFD-fed mice. We showed that diet-induced obesity leads to increased miRNAs content of AT-Exos mainly enriched in lipid metabolism and inflammatory pathways associated with metabolic disorders. Exosomes derived from adipose tissue, either from the adipocytes, ATMs, or other stromal cells, have the capacity to modulate the functions of recipient cells. Our data indicated that AT-Exos from different depots exert distinct effects on adipocytes metabolism and inflammation. Particularly, vWAT-Exos appear to be key participant to lipid accumulation and inflammation in the development of obesity. miR-200a-3p and miR-200b-3p, act as pro-obesity factors in vWAT-Exos, promote the lipid accumulation in adipocytes partially via PI3K/AKT/mTOR pathway. Furthermore, targeting the SIRPα-CD47 axis can attenuate vWAT-Exos-induced metabolic disorders, providing insights into the development of potential therapeutic strategies for the future treatment of obesity and metabolic disorders.

## Supplementary Information

Additional file 1.

## Data Availability

MS lipidomics datasets have been deposited with MetaboLights as entry ID MTBLS10004. Raw miRNA sequencing data has been deposited with GEO as entry ID GSE264481. Raw RNA sequencing data has been deposited with GEO as entry ID GSE264480. Any additional information required to reanalyze the data reported in this paper is available from the lead contacts upon request, please contact dlw@smmu.edu.cn.

## Abbreviations

SIRPα: Signaling regulatory protein α
vWAT: Visceral white adipose tissue
iWAT: Inguinal white adipose tissue
SAT: Subcutaneous adipose tissue
BAT: Brown Adipose Tissue
IR: Insulin resistance
ATMs: Adipose tissue macrophages
DCs: Dendritic cells
RBCs: Red blood cells
BMDMs: Bone marrow-derived macrophages
M-CSF: Macrophage Colony Stimulating Factor 1
DMEM: Dulbecco’s Modified Eagle Medium
MEM-α: Minimum Essential Medium Alpha
IPGTT: Intraperitoneal glucose tolerance test
IPITT: Intraperitoneal insulin tolerance test
LC–MS: Liquid chromatography-mass spectrometry
DAPI: 4',6-Diamidino-2-phenylindole dihydrochloride
DAB: 3,3’-Diaminobenzidine
PLS-DA: Partial Least-Squares Discriminant Analysis
Zeb2: Zinc finger E-box binding homeobox 2
Slc23a2: Solute carrier family 23 member 2
IRS2: Insulin receptor substrate 2
VDR: Vitamin D receptor
NF-kB: Nuclear factor-kappaB
JNK: C-jun N-terminal kinase
MAPKs: Mitogen-activated protein kinases
Acc1: Acetyl-CoA carboxylase
Ucp1: Uncoupling protein 1
Fasn: Fatty acid synthase
Lpl: Lipoprotein lipase
PPARγ: Peroxisome proliferator-activated receptor γ
Cpt1a: Carnitine palmitoyltransferase 1a
Chrebp: Carbohydrate-responsive element-binding protein
PPARα: Peroxisome proliferator-activated receptor-α
ADSCs: Adipose stem cells
Cxcl1: C-X-C motif chemokine ligand 1
Cxcl2: C-X-C motif chemokine ligand 2
Cxcr2: C-X-C motif chemokine receptor 2
Ccr7: C–C motif chemokine receptor 7
