# Supplementary material for: Blocking the SIRPα-CD47 axis promotes macrophage phagocytosis of exosomes derived from visceral adipose tissue and improves inflammation and metabolism in mice
Source: J Biomed Sci. 2025 Feb 28;32:31. doi: 10.1186/s12929-025-01124-y (PMC11869713; doi:10.1186/s12929-025-01124-y)
Supplement: Supplementary file 1 — Additional file 1. [file 12929_2025_1124_MOESM1_ESM.docx]

**
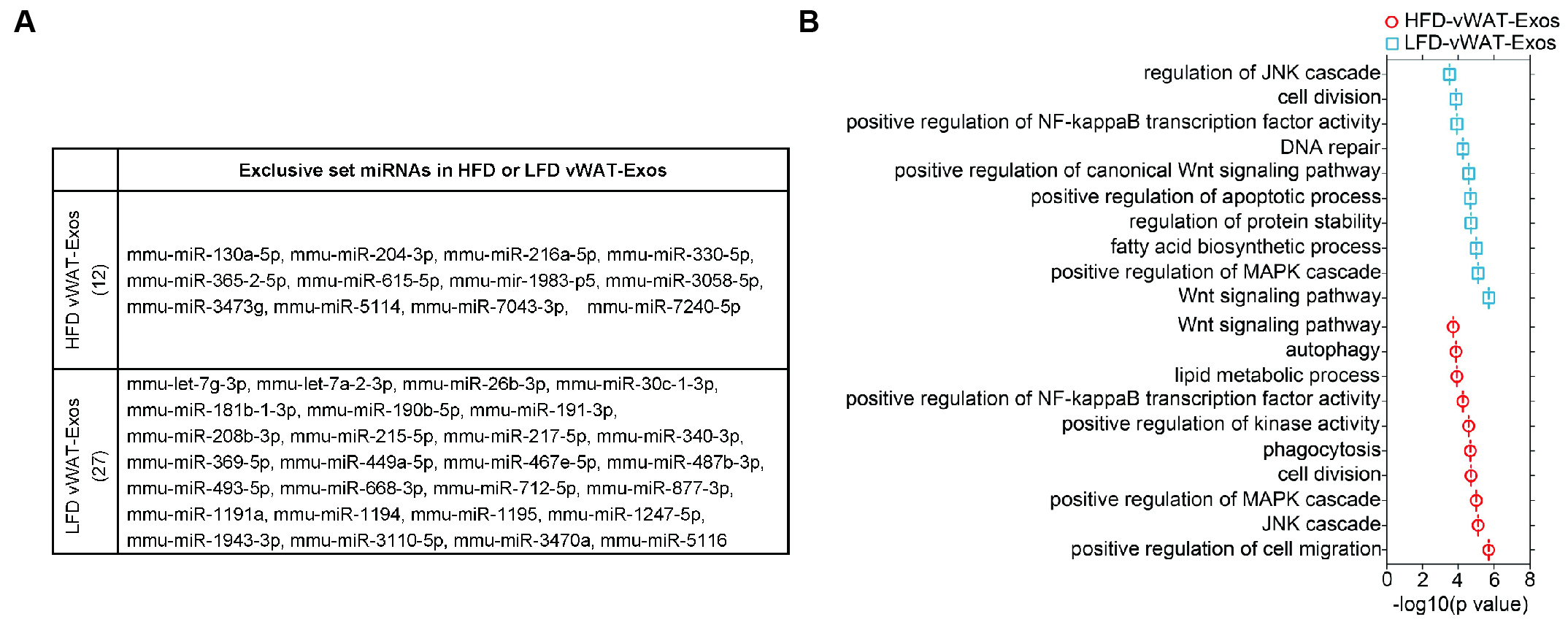
**

**Supplementary Fig. S1. Diet-induced obesity affects miRNAs composition of vWAT-Exos.**

**A** “Exclusive set” miRNAs in vWAT-Exos derived from HFD-fed and LFD-fed mice are displayed. **B** Column diagram shows the representative enriched GO-terms of predicted target genes for “exclusive set” miRNAs in vWAT-Exos derived from HFD-fed and LFD-fed mice

**
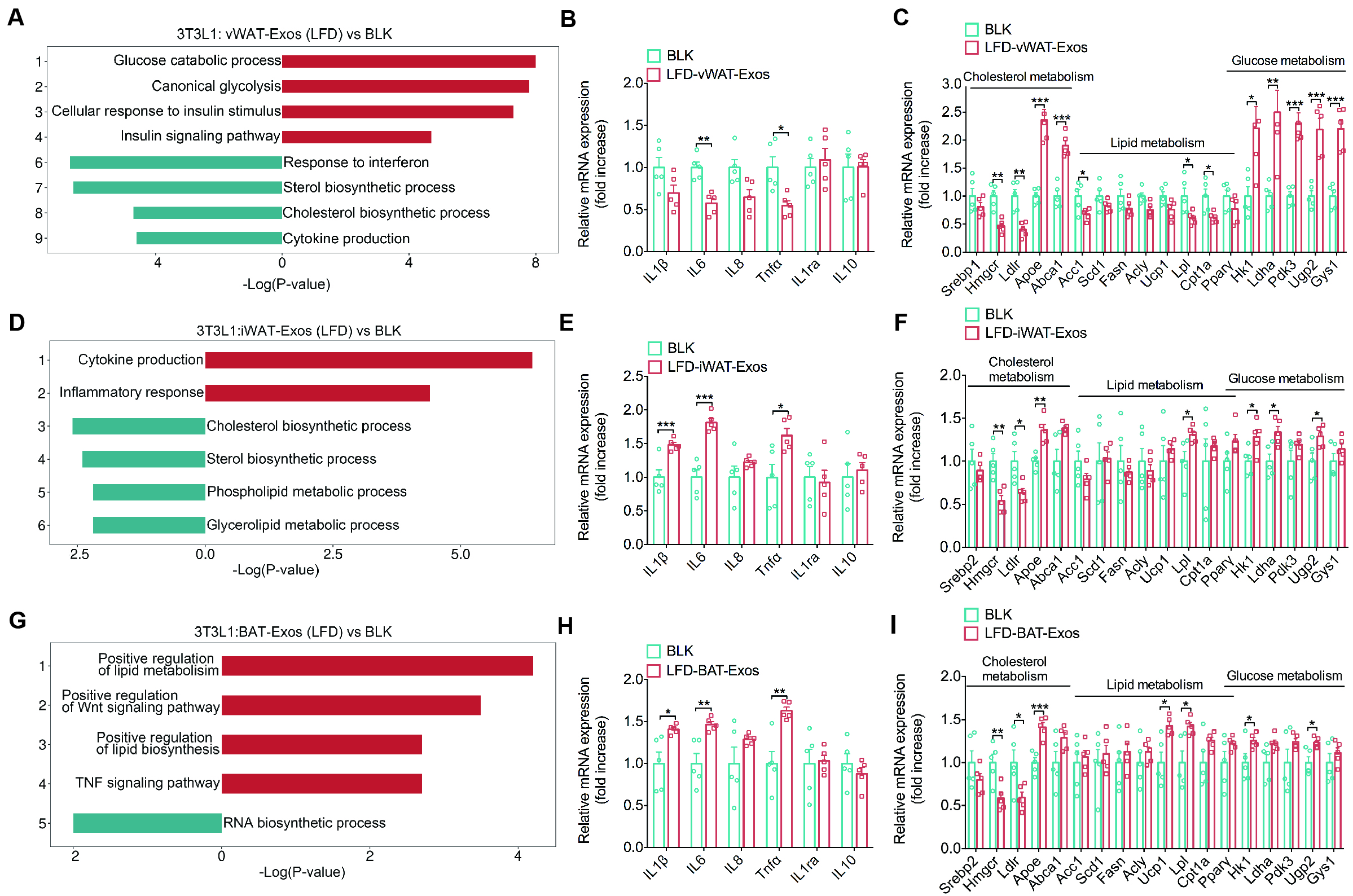
**

**Supplementary Fig. S2. The effects of AT-Exos derived from LFD-fed mice on mature 3T3L1 adipocytes. A** Top GO terms of the differentially expressed genes of 3T3L1 treated with LFD-vWAT-Exos and PBS buffer based on RNA-seq analysis. Red box and blue box represent up-regulated and down-regulated GO terms, respectively. **B and C** Normalized expression of inflammatory cytokine genes (panel b) and glycolipid metabolism genes (panel c) in 3T3L1 treated with vWAT-Exos derived from LFD-fed mice and PBS buffer. n = 5 per group. **D** Top GO terms of the differentially expressed genes of 3T3L1 treated with LFD-iWAT-Exos and PBS buffer based on RNA-seq analysis. Red box and blue box represent up-regulated and down-regulated GO terms, respectively. **E and F** Normalized expression of inflammatory cytokine genes (panel e) and glycolipid metabolism genes (panel f) in 3T3L1 treated with iWAT-Exos derived from LFD-fed mice and PBS buffer. n = 5 per group. **G** Top GO terms of the differentially expressed genes of 3T3L1 treated with LFD-BAT-Exos and PBS buffer based on RNA-seq analysis. Red box and blue box represent up-regulated and down-regulated GO terms, respectively. **H and I** Normalized expression of inflammatory cytokine genes (panel h) and glycolipid metabolism genes (panel i) in 3T3L1 treated with BAT-Exos derived from LFD-fed mice and PBS buffer. n = 5 per group. Statistical analyses were performed by Student’s t test. All data are presented as mean ± SEM. *P < 0.05, **P < 0.01 and ***P < 0.001.


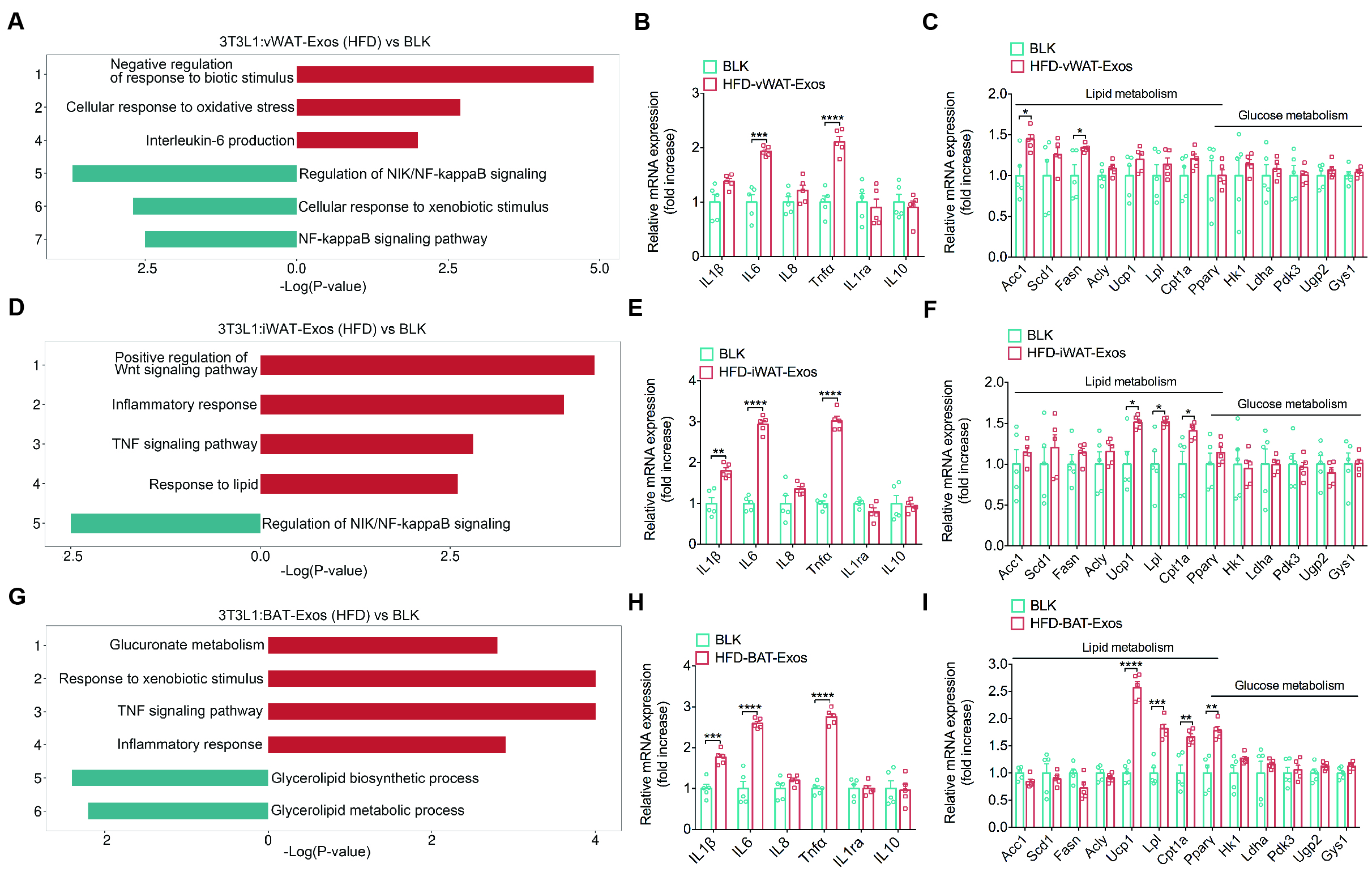


**Supplementary Fig. S3. The effects of AT-Exos from HFD-fed mice on mature 3T3L1 adipocytes.**

**A** Top GO terms of the differentially expressed genes of 3T3L1 treated with HFD-vWAT-Exos and PBS buffer based on RNA-seq analysis. Red box and blue box represent up-regulated and down-regulated GO terms, respectively. **B and C** Normalized expression of inflammatory cytokine genes (panel b) and glycolipid metabolism genes (panel c) in 3T3L1 treated with vWAT-Exos derived from HFD-fed mice and PBS buffer. n = 5 per group. **D** Top GO terms of the differentially expressed genes of 3T3L1 treated with HFD-iWAT-Exos and PBS buffer based on RNA-seq analysis. Red box and blue box represent up-regulated and down-regulated GO terms, respectively. **E and F** Normalized expression of inflammatory cytokine genes (panel e) and glycolipid metabolism genes (panel f) in 3T3L1 treated with iWAT-Exos derived from HFD-fed mice and PBS buffer. n = 5 per group. **G** Top GO terms of the differentially expressed genes of 3T3L1 treated with HFD-BAT-Exos and PBS buffer based on RNA-seq analysis. Red box and blue box represent up-regulated and down-regulated GO terms, respectively. **H and I** Normalized expression of inflammatory cytokine genes (panel h) and glycolipid metabolism genes (panel i) in 3T3L1 treated with BAT-Exos derived from HFD-fed mice and PBS buffer. n = 5 per group. Statistical analyses were performed by Student’s t test. All data are presented as mean ± SEM. *P < 0.05, **P < 0.01, ***P < 0.001 and ****P < 0.0001.


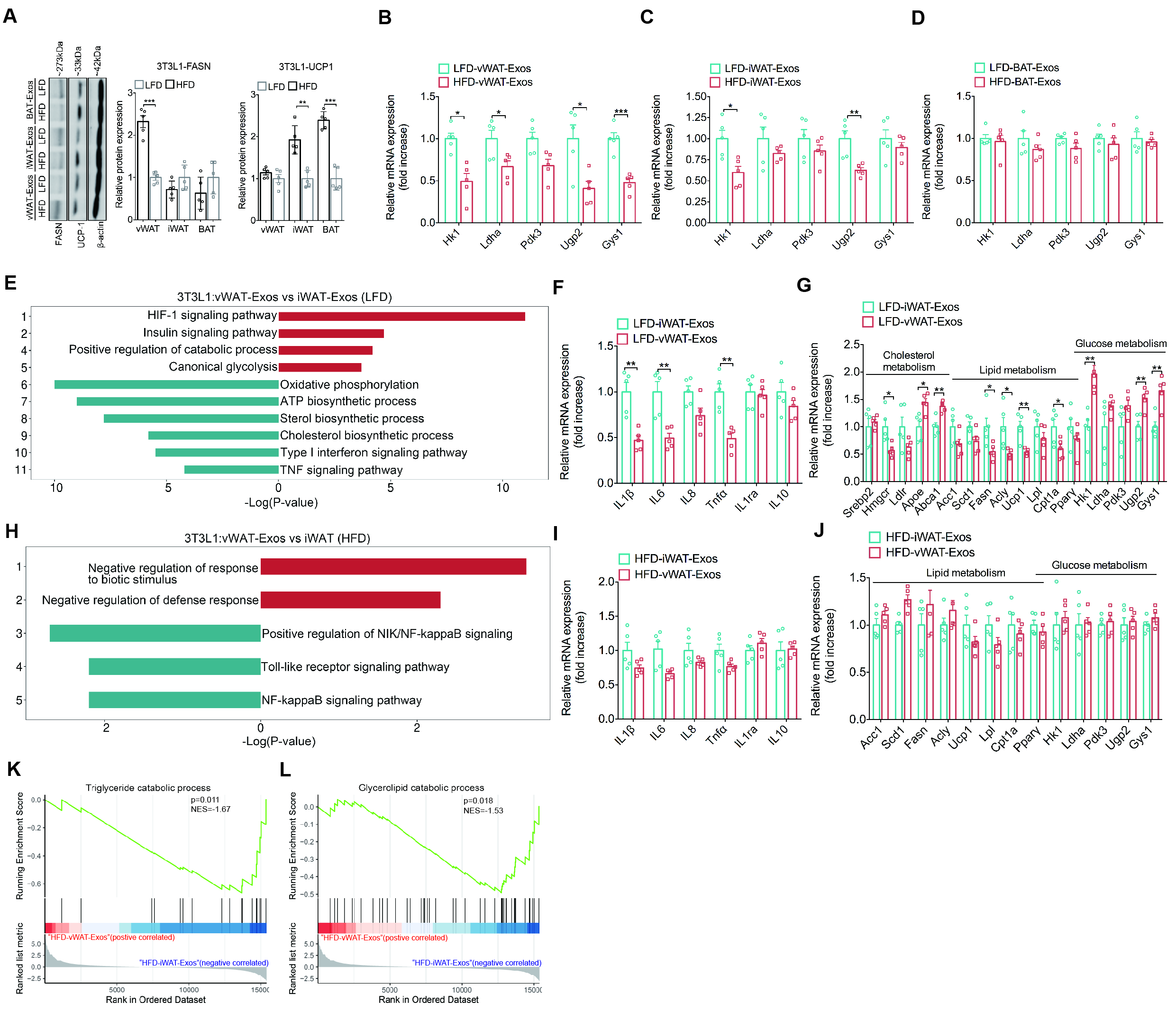


**Supplementary Fig. S4. Comparison of the effects that AT-Exos from HFD-fed and LFD-fed mice on mature 3T3L1 adipocytes.**

**A** Western blot analysis and quantification of FASN and UCP1 protein in 3T3L1 treated with AT-Exos derived from HFD-fed and LFD-fed mice. **B-D** Normalized expression of glucose metabolism-related genes in 3T3L1 treated with vWAT-Exos (panel b), iWAT-Exos (panel c) and BAT-Exos (panel d) derived from HFD-fed and LFD-fed mice. n = 5 per group. **E** Top GO terms of the differentially expressed genes of 3T3L1 treated with LFD-vWAT-Exos and LFD-iWAT-Exos based on RNA-seq analysis. Red box and blue box represent up-regulated and down-regulated GO terms, respectively. **F and G** Normalized expression of inflammatory cytokine genes (panel f) and glycolipid metabolism genes (panel g) in 3T3L1 treated with vWAT-Exos and iWAT-Exos derived from LFD-fed mice. n = 5 per group. **H** Top GO terms of the differentially expressed genes of 3T3L1 treated with HFD-vWAT-Exos and HFD-iWAT-Exos based on RNA-seq analysis. Red box and blue box represent up-regulated and down-regulated GO terms, respectively. **I and J** Normalized expression of inflammatory cytokine genes (panel i) and glycolipid metabolism genes (panel j) in 3T3L1 treated with vWAT-Exos and iWAT-Exos derived from LFD-fed mice. n = 5 per group. **K and L** GSEA analysis of differently expressed genes between HFD-vWAT-Exos and HFD-iWAT-Exos treatment group showing the triglyceride catabolic process (panel k) and glycerolipid catabolic process (panel l). NES: Normalized Enrichment Score. Statistical analyses were performed by Student’s t test. All data are presented as mean ± SEM. *P < 0.05, **P < 0.01 and ***P < 0.001.


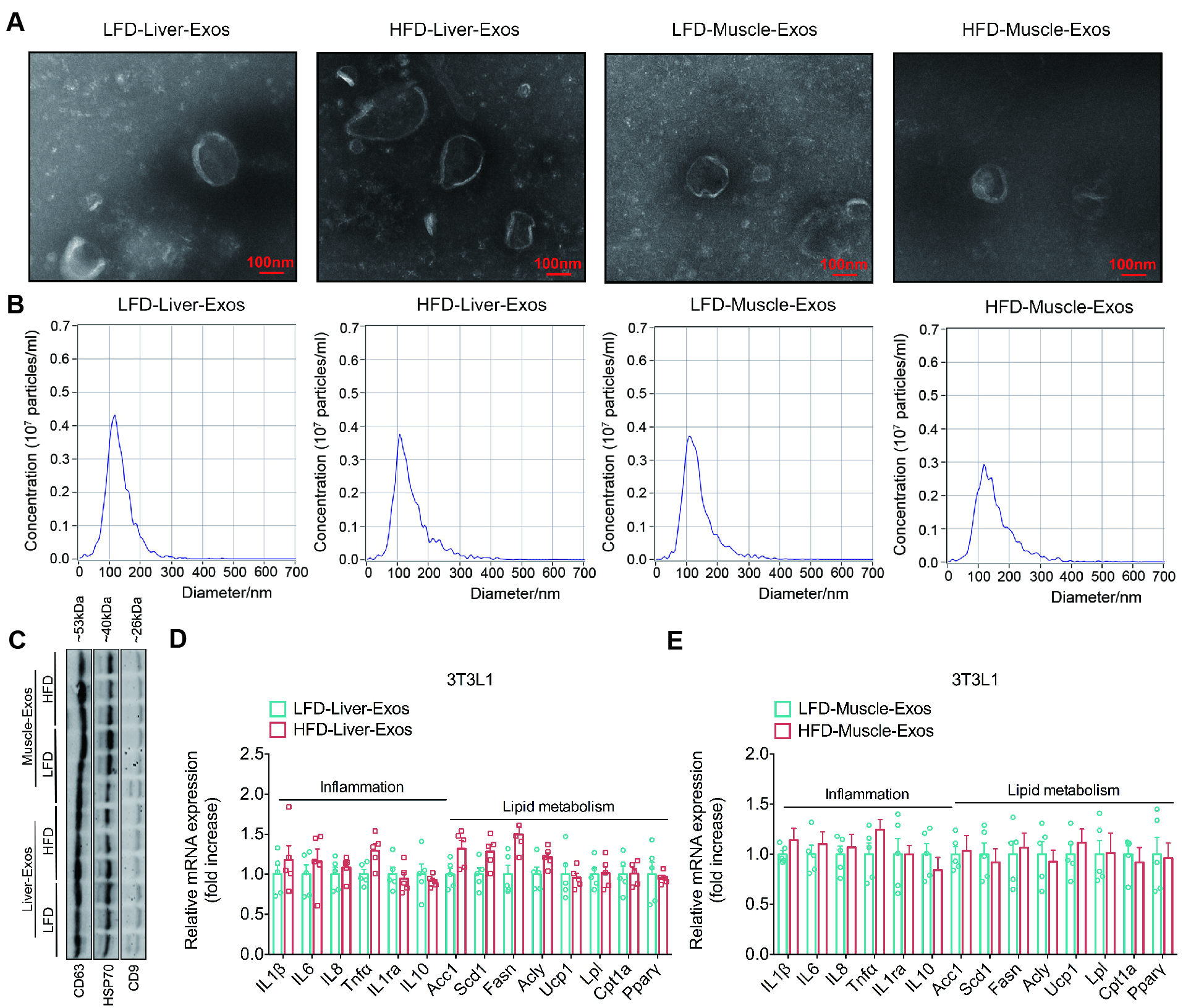


**Supplementary Fig. S5.** **No significant difference in adipocyte inflammation and lipid metabolism regulation by liver- or muscle-derived exosomes from HFD-fed and LFD-fed mice.**

**A** Representative electron microscopy images of exosomes. Scale bar, 100 nm. **B** The particle size of the Liver-Exos or Muscle-Exos measured by Nanoparticle tracking analysis. **C** The exosome-related protein markers CD63, HSP70 and CD9 measured by Western blot in Liver-Exos or Muscle-Exos. These blots are representative of three independent replicate experiments. **D and E** Normalized expression of inflammation-related and lipid metabolism-related genes in 3T3L1 treated with Liver-Exos (panel d) and Muscle-Exos (panel e) derived from HFD-fed and LFD-fed mice. n = 5 per group. Statistical analyses were performed by Student’s t test. All data are presented as mean ± SEM.


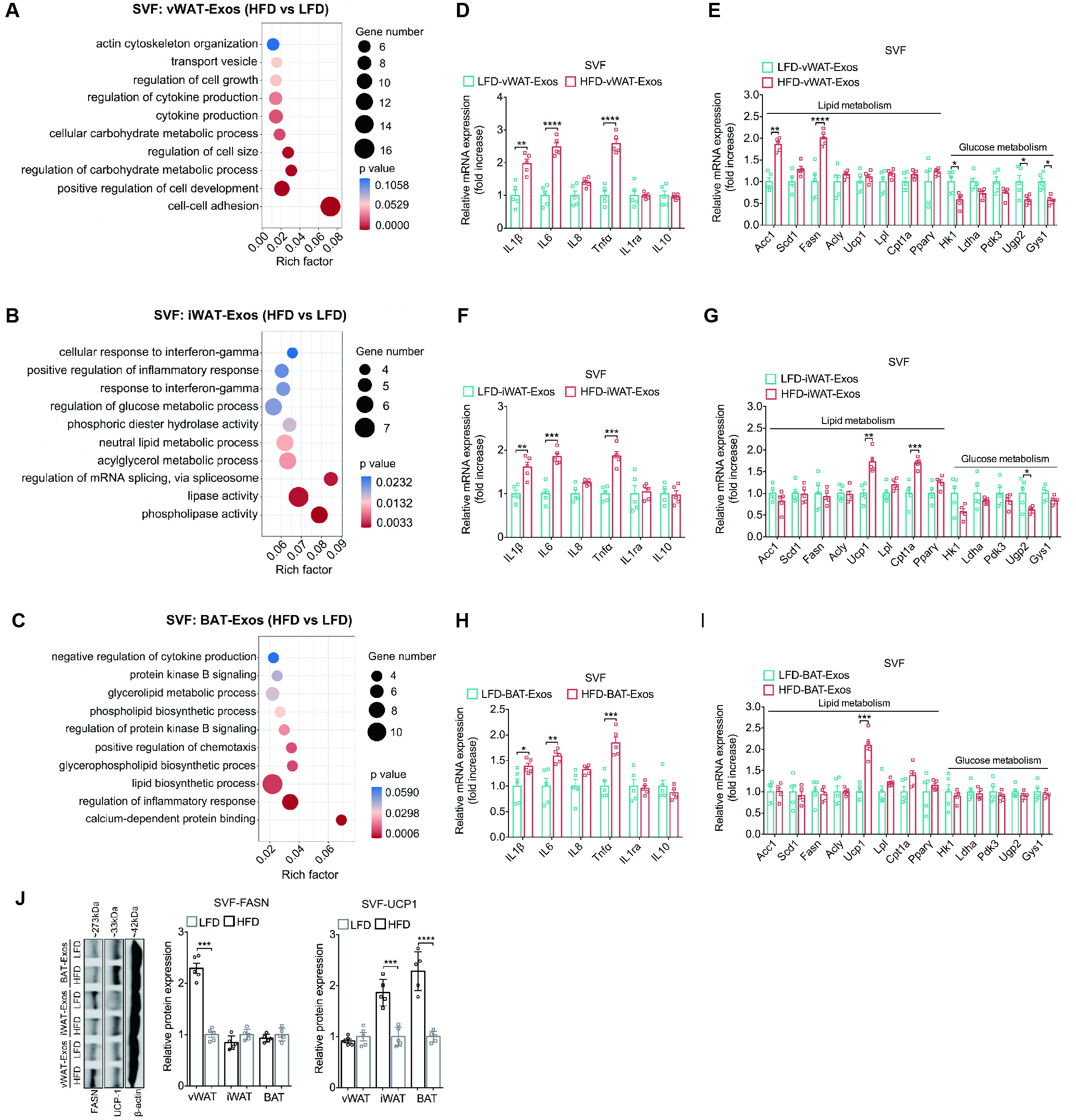


**Supplementary Fig. S6. AT-Exos from different depots participate in inflammation and metabolic homeostasis of SVF-adipocytes.**

**A-C** Top GO terms of the differentially expressed genes of SVF-adipocytes treated with vWAT-Exos (panel a), iWAT-Exos (panel b) and BAT-Exos (panel c) derived from HFD-fed and LFD-fed mice based on RNA-seq analysis. **D and E** Normalized expression of inflammation-related (panel d) and lipid metabolism-related genes (panel e) in SVF-adipocytes treated with vWAT-Exos derived from HFD-fed and LFD-fed mice. n = 5 per group. **F and G** Normalized expression of inflammation-related (panel f) and lipid metabolism-related genes (panel g) in SVF-adipocytes treated with iWAT-Exos derived from HFD-fed and LFD-fed mice. n = 5 per group. **H and I** Normalized expression of inflammation-related (panel h) and lipid metabolism-related genes (panel i) in SVF-adipocytes treated with BAT-Exos derived from HFD-fed and LFD-fed mice. n = 5 per group. **J** Western blot analysis and quantification of FASN and UCP1 protein in SVF-adipocytes treated with AT-Exos derived from HFD-fed and LFD-fed mice. Statistical analyses were performed by Student’s t test. All data are presented as mean ± SEM. *P < 0.05, **P < 0.01, ***P < 0.001 and ****P < 0.0001.


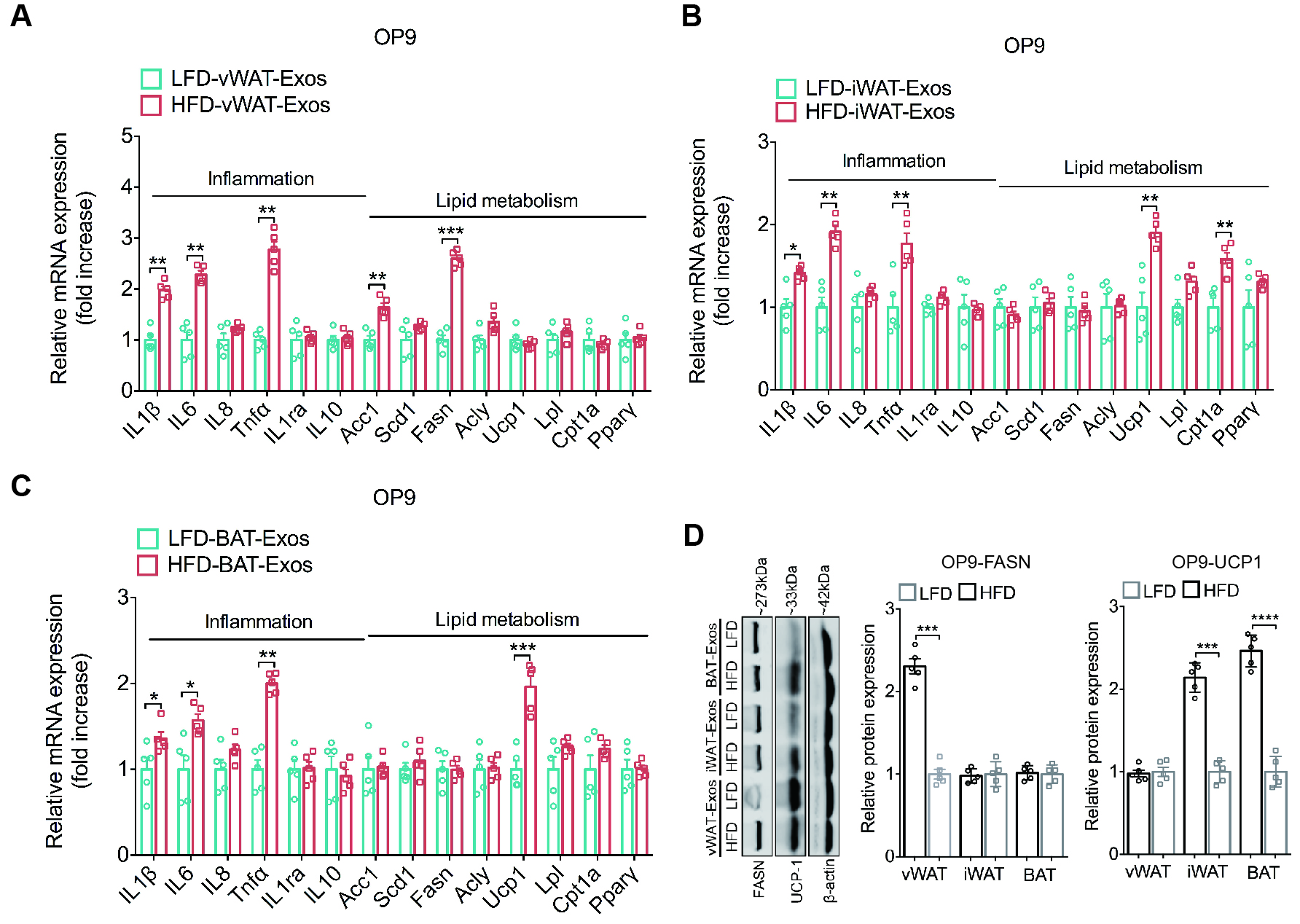


**Supplementary Fig. S7. AT-Exos from different depots participate in inflammation and metabolic homeostasis of OP9-adipocytes.**

**A-C** Normalized expression of inflammation-related and lipid metabolism-related genes in OP9-adipocytes treated with vWAT-Exos (panel a), iWAT-Exos (panel b) or BAT-Exos (panel c) derived from HFD-fed and LFD-fed mice. n = 5 per group. **D** Western blot analysis and quantification of FASN and UCP1 protein in OP9-adipocytes treated with AT-Exos derived from HFD-fed and LFD-fed mice. Statistical analyses were performed by Student’s t test. All data are presented as mean ± SEM. *P < 0.05, **P < 0.01 and ***P < 0.001.


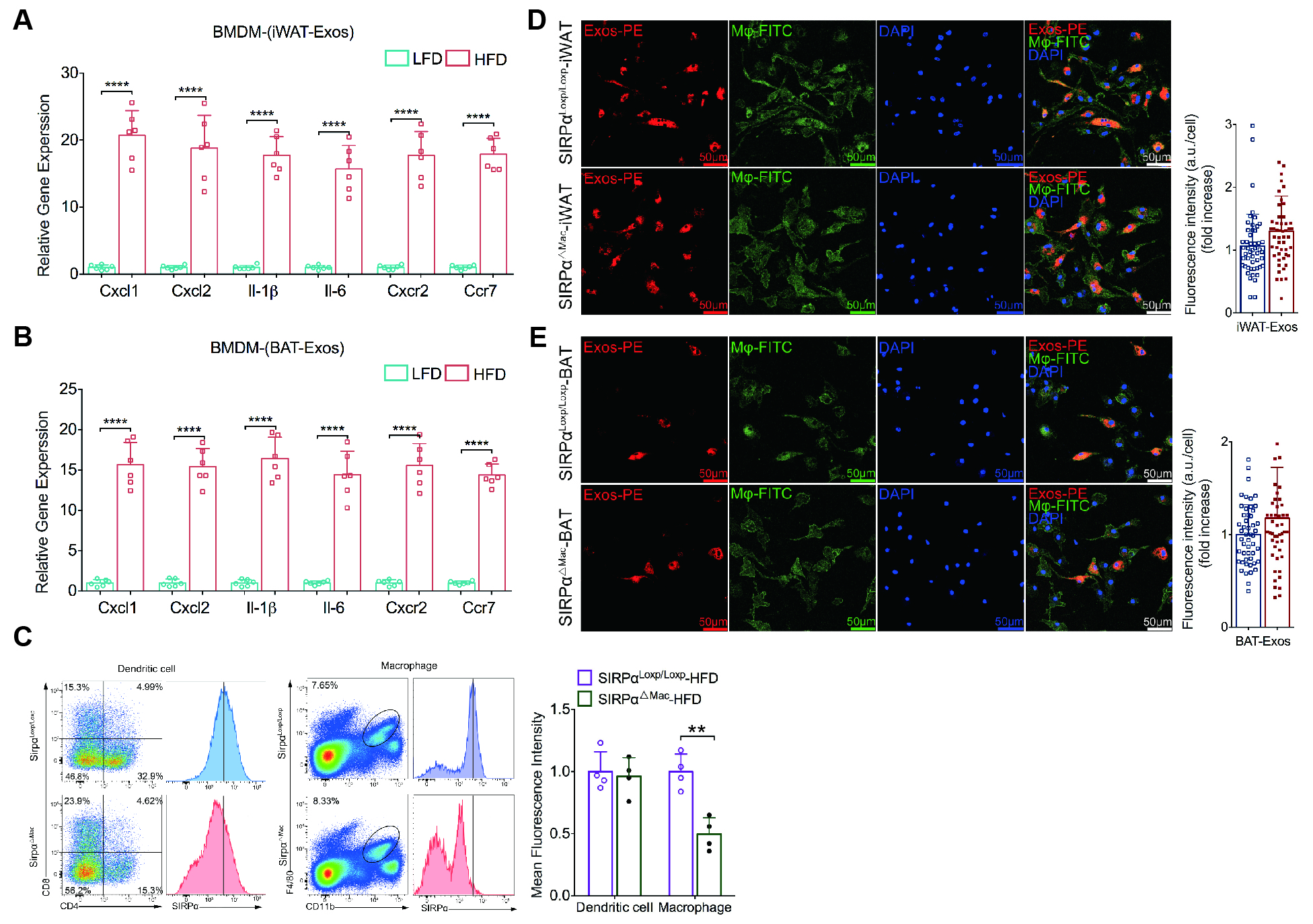


**Supplementary Fig. S8. iWAT-Exos or BAT-Exos from obese mice more effectively activated macrophages than those from lean individuals, yet macrophages showed no difference in phagocytic efficiency between obese and lean iWAT-Exos or BAT-Exos.**

**A and B** Normalized expression of inflammatory cytokine and chemokine genes in BMDMs treated with iWAT-Exos (panel a) or BAT-Exos (panel b) from HFD-fed and LFD-fed mice. n = 6 per group. **C** A representative image of flow cytometry analysis of SIRPα expression in splenic dendritic cells (left) and macrophages (middle) of SIRPα^ΔMac^ and SIRP^loxp/loxp^ mice, with the quantification of SIRPα expression was shown in right. n = 4 mice per group. **D and E** Representative confocal microscopy images showing macrophage (F4/80^+^ cells, green) derived from HFD-fed SIRPα^ΔMac^ and SIRPα^loxp/loxp^ mice phagocytosis for PKH26-labeled iWAT-Exos (panel d) and BAT-Exos (panel e) with quantitation shown in right. The cells nuclei were stained with DAPI (blue). n = 50 cells per group. Scale bar, 50 μm. Statistical analyses were performed by Student’s t test. All data are presented as mean ± SEM. *P < 0.05, **P < 0.01, ***P < 0.001 and ****P < 0.0001.


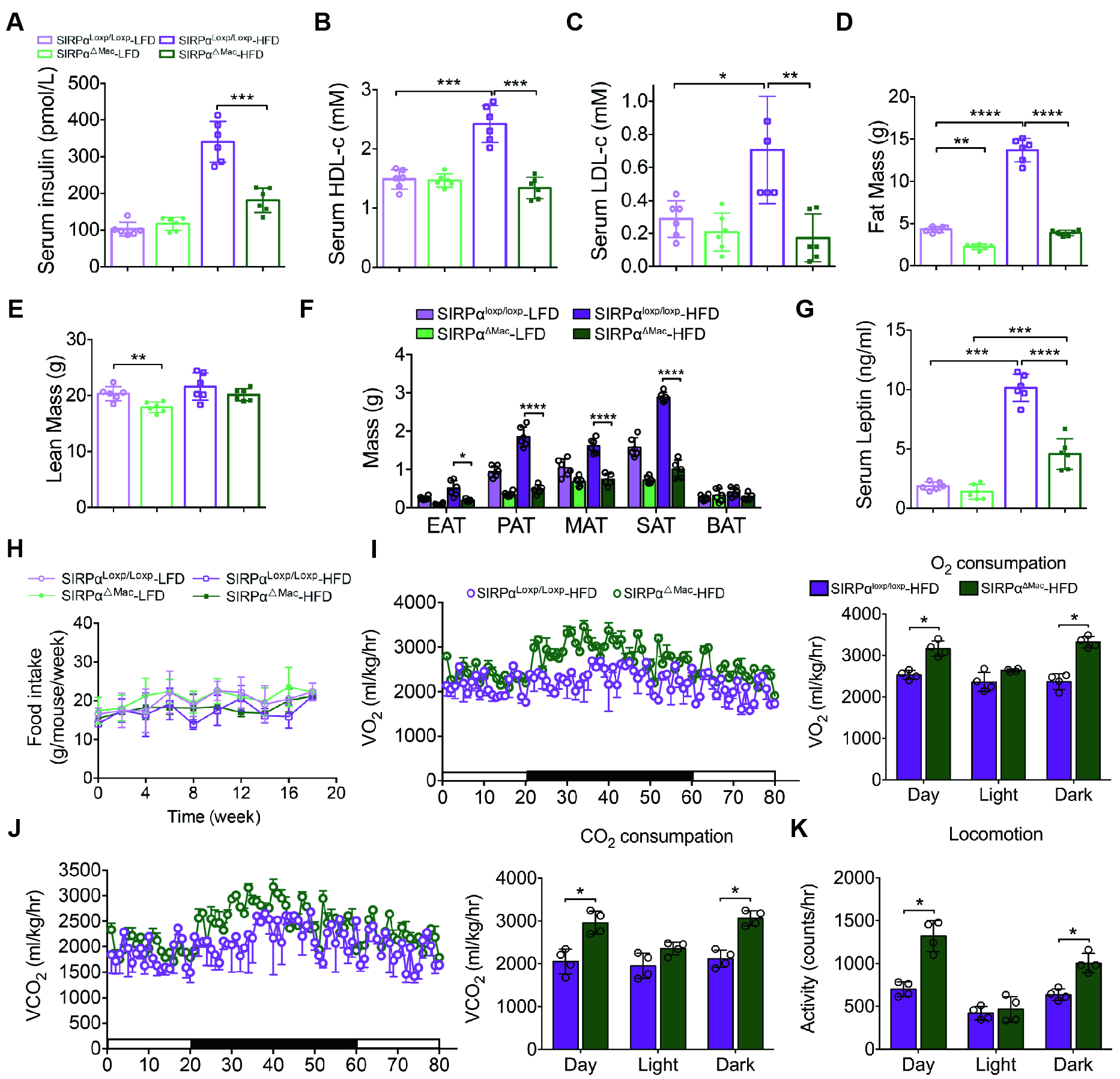


**Supplementary Fig. S9. SIRPα deficient mice treated with HFD show increased energy expenditure. A-C** Serum levels of insulin (panel a), HDL-c (panel b) and LDL-c (panel c) in SIRPα^ΔMac^ and SIRPα^loxp/loxp^ mice fed with LFD and HFD for 16 weeks. n = 6 mice per group. **D and E** Calculation of fat mass (panel d) and lean mass (panel e) of LFD-fed and HFD-fed SIRPα^ΔMac^ or SIRPα^loxp/loxp^ mice. n = 6 mice per group. **F** Weight of individual tissues including epididymal adipose tissue (EAT), perirenal adipose tissue (PAT), mesenteric adipose tissue (MAT), subcutaneous adipose tissue (SAT), brown adipose tissue (BAT) dissected from SIRPα^ΔMac^ and SIRPα^loxp/loxp^ mice following 16 weeks on LFD or HFD. n = 6 mice per group. **G** Serum levels of leptin in SIRPα^ΔMac^ and SIRPα^loxp/loxp^ mice fed with LFD and HFD for 16 weeks. n = 6 mice per group. **H** Food intake of chow diet. n = 9 mice per group. **I-K** Oxygen consumption (VO_2_) (panel i), carbon dioxide production (VCO_2_) (panel k) and locomotion activity (panel k) of SIRPα^ΔMac^ and SIRPα^loxp/loxp^ mice fed with LFD or HFD for 16 weeks. n = 4 mice per group. Statistical analyses were performed by Student’s t test (A-G, I-K) or one-way ANOVA followed by Tukey’s post hoc multiple-comparison test (H). All data are presented as mean ± SEM. *P < 0.05, **P < 0.01, ***P < 0.001 and ****P < 0.0001.


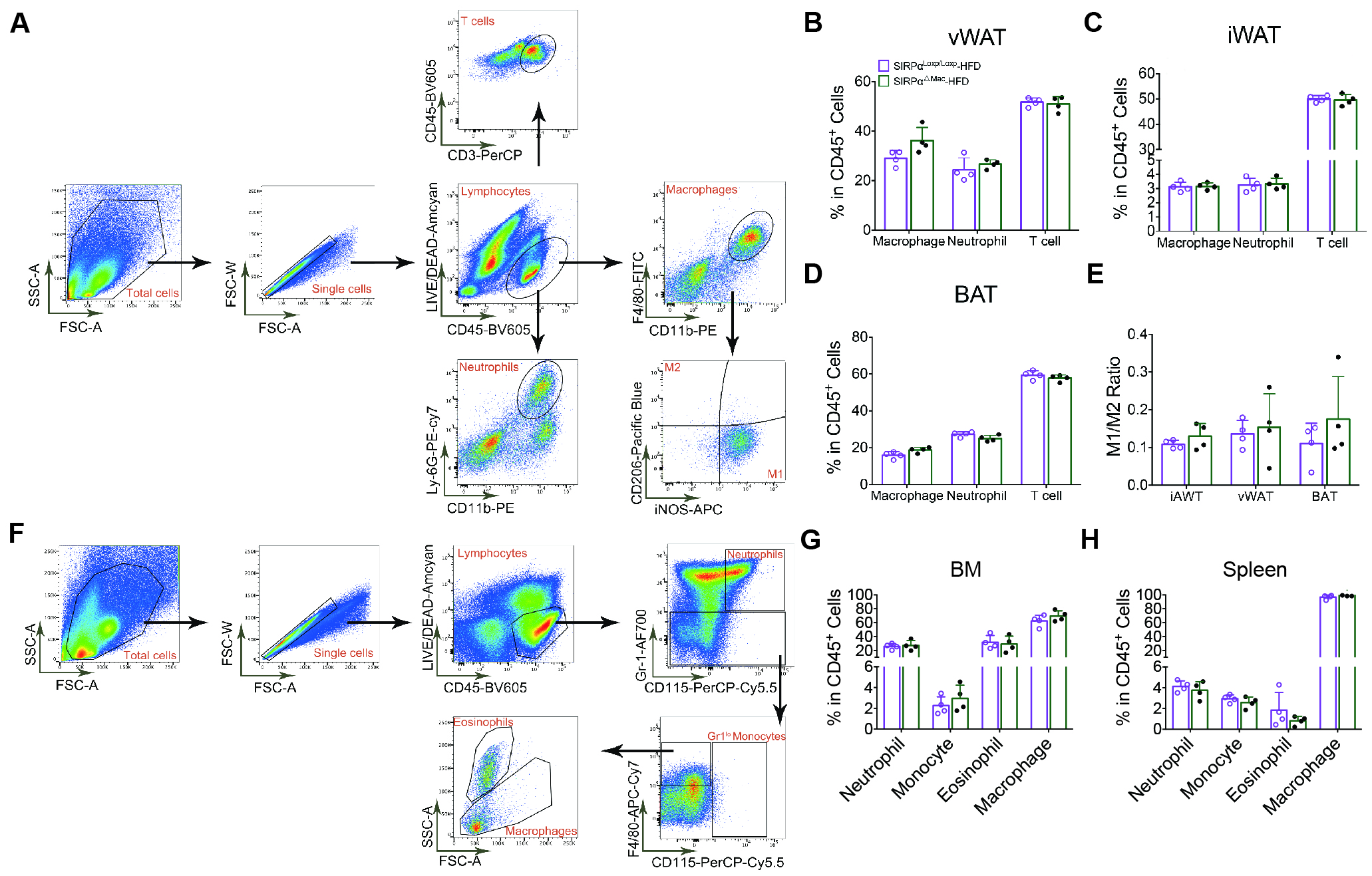


**Supplementary Fig. S10. SIRPα deletion in macrophages has minor impact on the amounts of inflammatory immune cells in adipose tissues, bone marrow and spleen under HFD conditions.**

**A** The gating strategy for detecting of immunocyte subsets in adipose tissue by flow cytometry. **B-D** The quantification of macrophage, neutrophil and T cells in vWAT (panel b), iWAT (panel c) and BAT (panel d) from SIRPα^ΔMac^ and SIRPα^loxp/loxp^ mice fed with HFD for 16 weeks. n = 4 mice per group. **E** The ratio of M1 and M2 macrophages in vWAT, iWAT and BAT. n = 4 per group. **F** The gating strategy for detecting of myeloid cell subsets in adipose tissue by flow cytometry. **G and H** The quantification of neutrophil, monocyte, eosinophil and macrophage in bone marrow (BM) (panel g) and spleen (panel h). n = 4 mice per group. Statistical analyses were performed by Student’s t test. All data are presented as mean ± SEM. *P < 0.05, **P < 0.01, ***P < 0.001 and ****P < 0.0001.


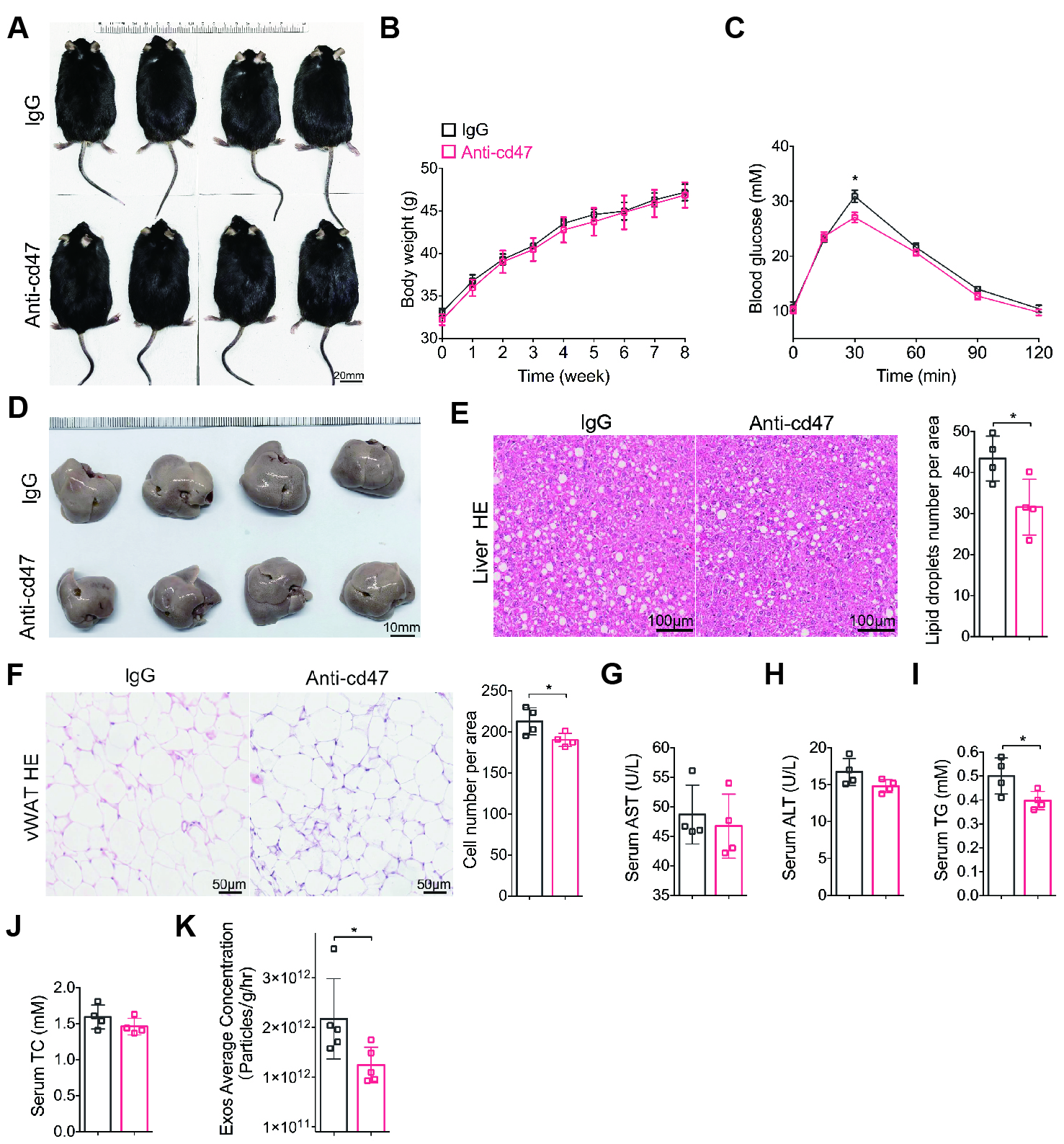


**Supplementary Fig. S11. Blocking the SIRPα-CD47 axis attenuates metabolic disorders of ob/ob mice.**

**A and B** Representative images of ob/ob mice (panel a). Animals were injected with lgG and neutralizing antibodies targeting CD47 for 8 weeks, with body weights were shown in (panel b). n = 4 mice per group. **C** IPGTT (2 g/kg) on those above mice. n = 4 mice per group. **D and E** Representative images of liver (panel d) and the HE staining of liver of ob/ob mice treated with lgG or neutralizing antibodies targeting CD47 for 8 weeks (panel e, left), and the quantification of lipid droplets number per area is shown (panel e, right). n = 4 mice per group. **F** Representative images of HE staining of vWAT sections of ob/ob mice treated with lgG or neutralizing antibodies targeting CD47 for 8 weeks (left), and the quantification of adipocytes number per area is shown in right. n = 4 mice per group. **G-J** Serum levels of AST (panel g), ALT (panel h), TG (panel i) and TC (panel j) in mice. n = 4 mice per group. **K** Quantification of vWAT-Exos released by per gram per hour adipose tissue from ob/ob mice treated with lgG or neutralizing antibodies targeting CD47 for 8 weeks. n = 4 per group. Statistical analyses were performed by Student’s t test. All data are presented as mean ± SEM. *P < 0.05.

**Supplementary Table S1. Primers for genotype mice.**

| Genotyping Primers | Primer sequences (5'to 3') |
| --- | --- |
| Sirpa^loxp/loxp^ mice | |
| Sirpα-1stloxPtF2 | CCGCTGCTCTGCTCAGGACTCGATT |
| Sirpα-1stloxPtR2 | CTCTGATGATCCTTTCTGGAACTTCACAC |
| Sirpα-2nd loxPtF2 | CCAAGAATCTCACAAAGAACACGGATG |
| Sirpα-2nd loxPtR2 | GACAGTGCTAAAGAGCTGTTGCCACG |
| Lyz-Cre mice | |
| Lyz-cre-Mutant | CCCAGAAATGCCAGATTACG |
| Lyz-cre-Common | CTTGGGCTGCCAGAATTTCTC |
| Lyz-cre-Wild type | TTACAGTCGGCCAGGCTGAC |

**Supplementary Table S2. Primers for real-time PCR**

| Gene | Forward Primer | Reverse Primer |
| --- | --- | --- |
| mIL1β | GCAACTGTTCCTGAACTCAACT | ATCTTTTGGGGTCCGTCAACT |
| mIL6 | TAGTCCTTCCTACCCCAATTTCC | TTGGTCCTTAGCCACTCCTTC |
| mIL8 | CAAGGCTGGTCCATGCTCC | TGCTATCACTTCCTTTCTGTTGC |
| mIL17a | TTTAACTCCCTTGGCGCAAAA | CTTTCCCTCCGCATTGACAC |
| mIL23 | ATGCTGGATTGCAGAGCAGTA | ACGGGGCACATTATTTTTAGTCT |
| mMip1a | TTCTCTGTACCATGACACTCTGC | CGTGGAATCTTCCGGCTGTAG |
| mAdiponectin | TGTTCCTCTTAATCCTGCCCA | CCAACCTGCACAAGTTCCCTT |
| mIFNγ | TCTGGAGGAACTGGCAAAAG | AGTGACAGGCTGGGATGG |
| mTNFα | CCCTCACACTCAGATCATCTTCT | GCTACGACGTGGGCTACAG |
| mMCP1 | TTAAAAACCTGGATCGGAACCAA | GCATTAGCTTCAGATTTACGGGT |
| mIL1ra | GCTCATTGCTGGGTACTTACAA | CCAGACTTGGCACAAGACAGG |
| mIL4 | GGTCTCAACCCCCAGCTAGT | GCCGATGATCTCTCTCAAGTGAT |
| mIL10 | GCTCTTACTGACTGGCATGAG | CGCAGCTCTAGGAGCATGTG |
| mIL13 | CCTGGCTCTTGCTTGCCTT | GGTCTTGTGTGATGTTGCTCA |
| mCxcl1 | ACTGCACCCAAACCGAAGTC | TGGGGACACCTTTTAGCATCTT |
| mCxcl2 | CCAACCACCAGGCTACAGG | GCGTCACACTCAAGCTCTG |
| mCxcr2 | ATGCCCTCTATTCTGCCAGAT | GTGCTCCGGTTGTATAAGATGAC |
| mCcr7 | TGTACGAGTCGGTGTGCTTC | GGTAGGTATCCGTCATGGTCTTG |
| mSrebp1 | TGACCCGGCTATTCCGTGA | CTGGGCTGAGCAATACAGTTC |
| mHmgcr | AGCTTGCCCGAATTGTATGTG | TCTGTTGTGAACCATGTGACTTC |
| mLdlr | TGACTCAGACGAACAAGGCTG | ATCTAGGCAATCTCGGTCTCC |
| mApoe | CTGACAGGATGCCTAGCCG | CGCAGGTAATCCCAGAAGC |
| mAbca1 | GCTTGTTGGCCTCAGTTAAGG | GTAGCTCAGGCGTACAGAGAT |
| mAcc1 | GATGAACCATCTCCGTTGGC | GACCCAATTATGAATCGGGAGTG |
| mScd1 | TTCTTGCGATACACTCTGGTGC | CGGGATTGAATGTTCTTGTCGT |
| mFasn | GGAGGTGGTGATAGCCGGTAT | TGGGTAATCCATAGAGCCCAG |
| mAcly | ACCCTTTCACTGGGGATCACA | GACAGGGATCAGGATTTCCTTG |
| mUcp1 | GGGCATTCAGAGGCAAATCAGCTT | ACACTGCCACACCTCCAGTCATTA |
| mLpl | GGGAGTTTGGCTCCAGAGTTT | TGTGTCTTCAGGGGTCCTTAG |
| mCpt1a | CTCCGCCTGAGCCATGAAG | CACCAGTGATGATGCCATTCT |
| mCpt1b | GACTTCCGGCTTAGTCGGG | GAATAAGGCGTTTCTTCCAGGA |
| mPparα | AGAGCCCCATCTGTCCTCTC | ACTGGTAGTCTGCAAAACCAAA |
| mPparγ | AGGCGAGGGCGATCTTGACAG | AATTCGGATGGCCACCTCTTTG |
| mHk1 | CGGAATGGGGAGCCTTTGG | GCCTTCCTTATCCGTTTCAATGG |
| mLdha | TGTCTCCAGCAAAGACTACTGT | GACTGTACTTGACAATGTTGGGA |
| mPdk3 | TCCTGGACTTCGGAAGGGATA | GAAGGGCGGTTCAACAAGTTA |
| mUgp2 | AGCAAAGCTATGTCTCAAGATGG | GAGGCTGCTGTGGTAAGTATTT |
| mGys1 | GAACGCAGTGCTTTTCGAGG | CCAGATAGTAGTTGTCACCCCAT |
| mPgc1α | TATGGAGTGACATAGAGTGTGCT | GTCGCTACACCACTTCAATCC |
| mUcp3 | CTGCACCGCCAGATGAGTTT | ATCATGGCTTGAAATCGGACC |
| mPrdm16 | CCAAGGCAAGGGCGAAGAA | AGTCTGGTGGGATTGGAATGT |
| mCox7a1 | CAGCGTCATGGTCAGTCTGT | AGAAAACCGTGTGGCAGAGA |
| mFatp1 | CTGGGACTTCCGTGGACCT | TCTTGCAGACGATACGCAGAA |
| mSlc27a1 | CGCTTTCTGCGTATCGTCTG | GATGCACGGGATCGTGTCT |
| mCd40 | TGTCATCTGTGAAAAGGTGGTC | ACTGGAGCAGCGGTGTTATG |
| mCd137 | CGTGCAGAACTCCTGTGATAAC | GTCCACCTATGCTGGAGAAGG |
| mβ-actin | GGCTGTATTCCCCTCCATCG | CCAGTTGGTAACAATGCCATGT |
| m36B4 | GCCGTGATGCCCAGGGAAGA | CATCTGCTTGGAGCCCACGTT |
| U6 Forward | CTCGCTTCGGCAGCACA | |
| U6 Reverse | AACGCTTCACGAATTTGCGT | |
| miR-200a-3p | GCGCGTAACACTGTCTGGTAA | |
| miR-200b-3p | GCGCGTAATACTGCCTGGTAA | |
| miR-125a-5p | GCGTCCCTGAGACCCTTTAAC | |
| miR-455-3p | CGGCAGTCCACGGGCAT | |
| miR-125b-5p | CGCGTCCCTGAGACCCTAAC | |

**Supplementary Table S3. Lipids alteration in the HFD-vWAT group and LFD-vWAT group**

| **HFD-vWAT vs. LFD-vWAT** | | | |
| --- | --- | --- | --- |
| Lipid | VIP | p Value | FC |
| PS 18:1_22:6 (C02737^b^) | 3.43 | .00 | 15.38^a^ |
| PS 42:10 (C02737) | 3.34 | .00 | 7.85 |
| PS 18:1_20:4 (C02737) | 2.79 | .00 | 7.32 |
| PS 40:7 (C02737) | 3.08 | .00 | 6.79 |
| SM 8:0;2O/22:0 (C00550) | 3.49 | .00 | 12.55 |
| SM 9:0;2O/34:2 (C00550) | 3.23 | .00 | 8.17 |
| SM 9:0;2O/32:2 (C00550) | 3.02 | .00 | 8.12 |
| SM 43:2;2O (C00422) | 3.18 | .00 | 7.12 |
| SM 9:0;2O/34:3 (C00550) | 2.91 | .00 | 6.67 |
| SM 36:2;3O (C00550) | 2.77 | .00 | 6.18 |
| SM 45:3;3O (C00550); | 1.33 | .01 | 0.37 |
| PI 18:0_20:3 (C01194) | 3.27 | .00 | 9.76 |
| PI 38:3 (C01194) | 3.02 | .00 | 7.84 |
| PE O-16:1_20:3 (C04475) | 3.42 | .00 | 8.58 |
| PE O-16:1_20:5 (C04475) | 2.95 | .00 | 8.29 |
| PE O-16:1_16:1 (C04475) | 3.12 | .00 | 7.37 |
| PE O-17:1_22:6 (C04475) | 2.82 | .00 | 6.73 |
| PE 16:0_16:1 (C00350) | 2.99 | .00 | 6.39 |
| PE O-17:1_18:1 (C04475) | 3.13 | .00 | 6.33 |
| PC 11:0_19:1 (C00157) | 2.96 | .00 | 6.88 |
| PC 15:0_16:1 (C00157) | 3.04 | .00 | 6.65 |
| PC 20:3_20:4 (C00157) | 3.06 | .00 | 6.21 |
| PC 18:0_17:1 (C00157) | 3.05 | .00 | 6.21 |
| PC 16:0_17:1 (C00157) | 2.9 | .00 | 6.01 |
| PC 32:0 (C00157); | 1.7 | .01 | 0.43 |
| LPC 40:7-SN2 (C04230); | 1.66 | .00 | 0.39 |
| Cer 18:1;2O/18:3 (C00195); | 1.07 | .03 | 0.49 |
| SHexCer 31:4;3O (C06125); | 1.80 | .00 | 0.32 |
| PG 18:2_18:2 (C00344); | 1.93 | .00 | 0.32 |
| TG 12:0_16:1_18:1 (C00422) | 2.79 | .00 | 8.37 |
| TG 16:0_17:1_18:1 (C00422) | 2.69 | .00 | 7.27 |
| TG 14:0_16:0_16:1 (C00422) | 2.47 | .00 | 6.95 |
| TG 14:0_16:1_18:1 (C00422) | 2.44 | .00 | 6.57 |
| TG 16:0_18:1_19:1 (C00422) | 2.60 | .00 | 6.57 |
| TG 16:0_16:0_17:1 (C00422) | 2.57 | .00 | 6.52 |
| TG 15:0_18:1_18:1 (C00422) | 2.68 | .00 | 6.25 |
| TG 18:1_18:5_19:1 (C00422); | 1.15 | .03 | 0.41 |
| TG 8:0_8:0_18:1 (C00422); | 1.94 | .00 | 0.22 |
| TG 16:2_18:5_19:2 (C00422); | 2.6 | .00 | 0.20 |
| TG 10:0_10:0_12:0 (C00422). | 2.61 | .00 | 1.90 |

Abbreviations: FC, fold change; PS, Phosphatidylserine; SM, Sphingomyelin; PI, Phosphatidylinositol; PE, Plasmanylethanolamine; PC, Phosphatidylcholine; LPC, Lysophosphatidylcholine; Cer, Ceramide; SHexCer, Cerebroside 3-sulfate; PG, Phosphatidylglycerol; TG, Triacylglycerol; VIP, variable important inprojection.

a：Lipids pass the threshold of VIP>1, p<.05, FC>6.00 or<.50 of HFD-vWAT versus LFD-vWAT

b：KEGG database number

**Supplementary Table S4. Lipids alteration in the HFD-BAT group and LFD-BAT group**

| **HFD-BAT VS LFD-BAT** | | | |
| --- | --- | --- | --- |
| Lipid | VIP | p Value | FC |
| PI 38:3 (C01194^b^) | 3.76 | .00 | 9.94^a^ |
| PI 18:0_20:3 (C01194) | 2.87 | .00 | 6.67 |
| PE O-16:1_20:5 (C04475) | 3.12 | .00 | 9.08 |
| PC 15:1_20:4 (C00157) | 2.75 | .00 | 7.44 |
| PC 15:0_16:0 (C00157) | 2.84 | .00 | 7.20 |
| LPC 15:1-SN1 (C04230) | 3.17 | .00 | 6.16 |
| LPC 40:7-SN2 (C04230) | 2.85 | .00 | 0.30 |
| SM 42:7;3O (C00550) | 1.90 | .00 | 0.43 |
| SM 45:2;3O (C00550) | 1.39 | .01 | 0.38 |
| TG 10:0_16:0_18:1 (C00422) | 2.34 | .03 | 8.25 |
| TG 16:0_17:0_18:1 (C00422) | 2.54 | .00 | 8.13 |
| TG 14:0_16:0_16:1 (C00422) | 2.47 | .04 | 7.71 |
| TG 16:0_16:0_17:1 (C00422) | 2.47 | .00 | 7.21 |
| TG 16:0_17:1_18:1 (C00422) | 2.40 | .01 | 6.71 |
| TG 12:0_16:0_18:1 (C00422) | 2.56 | .00 | 6.66 |
| TG 16:0_18:1_19:1 (C00422) | 2.48 | .01 | 6.62 |
| TG 12:0_16:0_16:1 (C00422) | 2.42 | .00 | 6.04 |
| TG 10:0_10:0_12:0 (C00422) | 2.20 | .04 | 0.34 |
| TG 9:0_18:1_28:7 (C00422) | 1.75 | .02 | 0.29 |
| TG 16:2_18:5_19:2 (C00422) | 2.62 | .00 | 0.25 |
| TG 8:0_8:0_18:1 (C00422) | 2.37 | .00 | 0.20 |

Abbreviations: FC, fold change; PI, Phosphatidylinositol; PE, Plasmanylethanolamine; PC, Phosphatidylcholine; LPC, Lysophosphatidylcholine; SM, Sphingomyelin; TG, Triacylglycerol; VIP, variable important inprojection.

a：Lipids pass the threshold of VIP>1, p<.05, FC>6.00 or<.50 of HFD-BAT versus LFD-BAT

b：KEGG database number

**Supplementary Table S5. Lipids alteration in the LFD-vWAT group and LFD-BAT group**

| **LFD-vWAT VS LFD-BAT** | | | |
| --- | --- | --- | --- |
| Lipid | VIP | p Value | FC |
| PC 35:2 (C00157^b^) | 2.75 | .00 | 11.55^a^ |
| PC O-37:1 (C00157) | 2.62 | .00 | 7.83 |
| PC 15:0_16:0 (C00157) | 1.99 | .00 | 7.17 |
| PC 16:0_16:0 (C00157) | 2.29 | .00 | 6.92 |
| PC O-16:0_16:0 (C05212 ) | 2.24 | .00 | 6.15 |
| PC O-39:3 (C05212 ) | 2.19 | .00 | 6.14 |
| PS 18:1_18:1 (C02737) | 2.90 | .00 | 11.23 |
| PS 18:0_22:6 (C02737) | 2.59 | .00 | 11.16 |
| PS 18:0_20:4 (C02737) | 2.68 | .00 | 8.39 |
| PS 18:0_22:4 (C02737) | 2.51 | .00 | 8.05 |
| PS 18:0_18:1 (C02737) | 2.38 | .00 | 7.49 |
| LPC 32:0-SN1 (C04230) | 2.43 | .00 | 10.89 |
| LPC 34:1-SN1 (C04230) | 2.18 | .00 | 7.48 |
| LPC 36:4-SN1 (C04230) | 2.26 | .01 | 6.74 |
| LPC 22:6-SN1 (C04230) | 1.08 | .00 | 0.49 |
| LPC 32:7-SN2 (C04230) | 1.30 | .00 | 0.45 |
| LPC 18:2 (C04230) | 1.83 | .00 | 0.44 |
| LPC 18:2-SN1 (C04230) | 1.85 | .00 | 0.41 |
| PE O-16:1_20:4 (C04475) | 2.58 | .00 | 6.55 |
| PE O-18:1_20:4 (C04475) | 2.47 | .00 | 6.49 |
| PE O-17:1_22:6 (C04475) | 2.23 | .00 | 6.49 |
| PE O-16:1_22:4 (C04475) | 2.29 | .00 | 6.16 |
| CAR 18:0 (C02301) | 2.68 | .00 | 8.28 |
| Cer 21:2;2O/21:1 (C00195) | 2.53 | .00 | 8.23 |
| Cer 17:1;2O/16:0 (C00195) | 2.44 | .00 | 6.78 |
| Cer 18:1;2O/24:1 (C00195) | 2.38 | .00 | 6.39 |
| SHexCer 31:4;3O (C06125) | 1.48 | .00 | 0.39 |
| PE-Cer 13:2;2O/21:2 (C06062) | 1.16 | .02 | 0.43 |
| SM 8:0;2O/34:3 (C00550) | 2.31 | .00 | 8.17 |
| SM 38:4;3O (C00550) | 2.18 | .00 | 6.12 |
| SM 34:1;3O (C00550) | 2.14 | .00 | 6.01 |
| TG 17:2_20:4_20:4 (C00422) | 1.44 | .04 | 0.35 |

Abbreviations: FC, fold change; PC, Phosphatidylcholine; PS, Phosphatidylserine; LPC, Lysophosphatidylcholine; PE, Plasmanylethanolamine; CAR, O-Acylcarnitine; Cer, Ceramide; SHexCer, Cerebroside 3-sulfate; PE-Cer, Ceramide phosphoethanolamine; SM, Sphingomyelin; TG, Triacylglycerol; VIP, variable important inprojection.

a：Lipids pass the threshold of VIP>1, p<.05, FC>6.00 or<.50 of LFD-WAT versus LFD-BAT

b：KEGG database number

**Supplementary Table S6. Lipids alteration in the HFD-vWAT group and HFD-BAT group**

| **HFD-vWAT VS HFD-BAT** | | | |
| --- | --- | --- | --- |
| Lipid | VIP | p Value | FC |
| PS 18:0_22:6 (C02737^b^) | 2.83 | .00 | 9.82^a^ |
| PS 18:0_22:5 (C02737) | 2.40 | .00 | 7.64 |
| PS 18:0_18:1 (C02737) | 2.48 | .00 | 6.99 |
| PS 18:1_18:1 (C02737) | 2.41 | .00 | 6.37 |
| SM 8:0;2O/34:3 (C00550) | 2.69 | .00 | 9.37 |
| SM 34:1;3O (C00550) | 2.70 | .00 | 8.05 |
| SM 36:4;3O (C00550) | 2.75 | .00 | 7.98 |
| SM 8:0;2O/26:0 (C00550) | 2.69 | .00 | 7.69 |
| SM 33:1;2O (C00550) | 2.57 | .00 | 7.16 |
| SM 8:1;2O/25:0 (C00550) | 2.62 | .00 | 7.14 |
| SM 8:0;2O/27:0 (C00550) | 2.42 | .00 | 7.13 |
| SM 9:0;2O/32:2 (C00550) | 2.38 | .00 | 6.60 |
| SM 34:2;2O (C00550) | 2.59 | .00 | 6.59 |
| SM 8:0;2O/28:2 (C00550) | 2.50 | .00 | 6.21 |
| SM 34:2;3O (C00550) | 2.47 | .00 | 6.18 |
| SM 42:3;3O (C00550) | 2.38 | .00 | 6.11 |
| SM 8:1;2O/29:0 (C00550) | 2.44 | .00 | 6.04 |
| SM 42:2;3O (C00550) | 2.37 | .00 | 6.01 |
| SM 40:3;3O (C00550) | 1.03 | .04 | 0.42 |
| LPC 32:0-SN1 (C04230) | 2.21 | .00 | 8.65 |
| LPC 34:1-SN1 (C04230) | 2.27 | .00 | 7.89 |
| LPC 30:0-SN1 (C04230) | 2.01 | .00 | 6.05 |
| LPC 20:4 (C04230) | 1.66 | .04 | 0.30 |
| LPC 22:6 (C04230) | 2.07 | .00 | 0.19 |
| LPC 22:6-SN1 (C04230) | 2.02 | .01 | 0.18 |
| PE O-17:1_20:4 (C04475) | 2.38 | .00 | 8.03 |
| PE O-16:1_18:1 (C04475) | 2.44 | .00 | 6.31 |
| PE O-18:2_22:6 (C04475) | 2.18 | .00 | 6.13 |
| PE 42:10 (C00350) | 1.48 | .01 | 0.42 |
| CAR 18:0 (C02301) | 2.44 | .00 | 7.48 |
| Cer 18:1;2O/24:1 (C00195) | 2.58 | .00 | 6.80 |
| PC O-39:3 (C05212 ) | 2.41 | .00 | 6.64 |
| PC 15:0_16:0 (C00157) | 2.16 | .00 | 6.50 |
| PI 18:0_20:4 (C01194) | 2.39 | .00 | 6.07 |
| SHexCer 31:4;3O (C06125) | 2.27 | .00 | 0.17 |

Abbreviations: FC, fold change; PS, Phosphatidylserine; SM, Sphingomyelin; LPC, Lysophosphatidylcholine; PE, Plasmanylethanolamine; CAR, O-Acylcarnitine; Cer, Ceramide; PC, Phosphatidylcholine; PI, Phosphatidylinositol; SHexCer, Cerebroside 3-sulfate; VIP, variable important inprojection.

a：Lipids pass the threshold of VIP>1, p<.05, FC>6.00 or<.50 of HFD-WAT versus HFD-BAT

b：KEGG database number
